# Radiation Induced One-Electron Oxidation of 2-Thiouracil in Aqueous Solutions

**DOI:** 10.3390/molecules24234402

**Published:** 2019-12-02

**Authors:** Konrad Skotnicki, Katarzyna Taras-Goslinska, Ireneusz Janik, Krzysztof Bobrowski

**Affiliations:** 1 Centre of Radiation Research and Technology, Institute of Nuclear Chemistry and Technology, 03-195 Warsaw, Poland; kris@ichtj.pl; 2 Faculty of Chemistry, Adam Mickiewicz University, 61-614 Poznan, Poland; karem@amu.edu.pl; 3 Notre Dame Radiation Laboratory, University of Notre Dame, Notre Dame, IN 46556, USA

**Keywords:** 2-thiouracil, radiosensitizers, ^●^OH and ^●^N_3_ radicals, 2c-3e S∴S-bonded intermediates, pulse radiolysis, TD-DFT methods, thiobases, nucleobase derivatives

## Abstract

Oxidative damage to 2-thiouracil (2-TU) by hydroxyl (^•^OH) and azide (^●^N_3_) radicals produces various primary reactive intermediates. Their optical absorption spectra and kinetic characteristics were studied by pulse radiolysis with UV-vis spectrophotometric and conductivity detection and by time-dependent density functional theory (TD-DFT) method. The transient absorption spectra recorded in the reactions of ^•^OH with 2-TU depend on the concentration of 2-TU, however, only slightly on pH. At low concentrations, they are characterized by a broad absorption band with a weakly pronounced maxima located at λ = 325, 340 and 385 nm, whereas for high concentrations, they are dominated by an absorption band with λ_max_ ≈ 425 nm. Based on calculations using TD-DFT method, the transient absorption spectra at low concentration of 2-TU were assigned to the ^●^OH-adducts to the double bond at C5 and C6 carbon atoms (3^●^, 4^●^) and 2c-3e bonded ^●^OH adduct to sulfur atom (1…^●^OH) and at high concentration of 2-TU also to the dimeric 2c-3e S-S-bonded radical in neutral form (2^●^). The dimeric radical (2^●^) is formed in the reaction of thiyl-type radical (6^●^) with 2-TU and both radicals are in an equilibrium with K_eq_ = 4.2 × 10^3^ M^−1^. Similar equilibrium (with K_eq_ = 4.3 × 10^3^ M^−1^) was found for pH above the pK_a_ of 2-TU which involves admittedly the same radical (6^●^) but with the dimeric 2c-3e S-S bonded radical in anionic form (2^●−^). In turn, ^●^N_3_-induced oxidation of 2-TU occurs via radical cation with maximum spin location on the sulfur atom which subsequently undergoes deprotonation at N1 atom leading again to thiyl-type radical (6^●^). This radical is a direct precursor of dimeric radical (2^●^).

## 1. Introduction

In 1988 George H. Hitchings and Gertrude B. Elion were awarded Nobel Prize in Physiology and Medicine for their groundbreaking work which laid the foundations for development of new drugs against a variety of diseases. Interestingly, their research in the 1950-s on the therapeutic properties of sulfur-substituted nucleobases (thiobases) resulted in two new chemotherapeutic drugs, thioguanine and 6-mercaptopurine, which were approved by US Food and Drug Administration (FDA) for treatment of acute leukemia and are still used for this purpose. Their revolutionary work was based on the fact, that substitution of carbonyl oxygen atom in canonical nucleobase by sulfur atom affects cell metabolism and leads to their death [1].

The aforementioned studies were devoted mostly to purine based thiobases but thiopyrimidines also have their place on the wide spectrum of biologically active compounds. First, they naturally occur in bacterial tRNA, up to date 11 different thiopyrimidine based compounds have been identified in bacteria [2], where they play important role in cellular metabolism [3]. There is also some evidence that thiobases play important role in metabolism of higher organism, for example, lack of certain sulfur-substituted nucleobases in mitochondria has been proven to lead to development of diabetes in mice [4].

Since replacement of oxygen atom by sulfur atom in DNA/RNA bases induces a substantial red-shift in absorption spectrum of the ground state, sulfur substituted nucleic acid bases are known photosensitive probes. Regular nucleobases absorb light mostly in ultra violet C (UVC) region (100–280 nm), while thiobases, depending on the structure absorb UVB (280–315 nm) and UVA (315–400 nm) radiation [5]. For this reason, 2-thiotymine is used for specific targeting of selected sites in nucleic acids [6]. What is even more important, thiobases (in contrast to canonical nucleobases) populate long-lived, reactive triplet state in high yields [7]. These properties are believed to be utilized in photodynamic therapy of various cancers. In fact, 4-thio-2-deoxythymidine has been proven to treat cancerous cell lines in vitro upon UVA irradiation [8,9] as well as skin cancer cells in biopsies [10,11] and bladder cancer in mice [12]. This compound have been currently moved to clinical trials [13]. For these reasons, it is not surprising that the vast majority of studies have been devoted to the photophysical and photochemical properties of thiobases related with their medical applications. There are several comprehensive research articles and reviews addressing these issues [14,15,16,17,18,19,20,21,22,23,24,25,26,27,28,29,30].

Moreover, sulfur substituted nucleobases were suggested to be good candidates for radiation therapy since they can potentially develop—similar to their halogenated analogues—radiosensitization properties [31]. The mechanism by which the former radiosensitizers operate was attributed to their reduction by thermalized/prehydrated [32] or ballistic electrons [33]. This results in formation of reactive radicals derived from the respective nucleobases which are believed to be responsible for DNA damage [34]. Therefore, it was highly desirable to understand how the sulfur-substituted nucleobases can be altered by the secondary electrons. A series of papers was published in recent five years addressing the dissociative electron attachment to 2-thiouracil (2-TU) and 2-thiothymine in gas-phase and showing that the fragmentation of these molecules arises mainly at the sulfur site [35,36,37,38,39,40]. Some attention was also paid to electron paramagnetic resonance (EPR) studies [41,42,43,44,45,46,47,48,49] combined with DFT calculations [50,51,52] of radicals derived from γ-irradiated single crystals of sulfur-substituted nucleobases.

Surprisingly, much less attention was directed into studies of radicals derived from sulfur nucleobases exposed to reactions with one-electron reductants and oxidants in aqueous phase. Reactions of hydrated electrons $e_{\mathrm{aq}}^{-}$ with bis(1-substituted-uracil-4-yl) disulfide [53], 2-thio analogues of cytosine and uracil [54], 8-thioguanosine [55], 5-iodo-4-thio-2′-deoxyuridine [56] and 5-bromo-4-thio-2′-deoxyuridine [57], H atoms (H^●^) with 8-thioguanosine [55], formate (${\mathrm{CO}}_{2}^{●-}$) and 2-hydroxypropan-2-yl ((CH_3_)_2_^●^COH) radicals with 2-thio analogues of cytosine and uracil [54], hydroxyl radicals (^•^OH), dichloride ( ${\mathrm{Cl}}_{2}^{●-}$) and dibromide ( ${\mathrm{Br}}_{2}^{●-}$) radical anions with 2-TU [58], 4-thiouracil [59] and 8-thioguanosine [55] and azide radicals (^●^N_3_) with 4-thiouracil [59] and 8-thioguanosine [55] are all but a few examples.

**Scheme 1:**
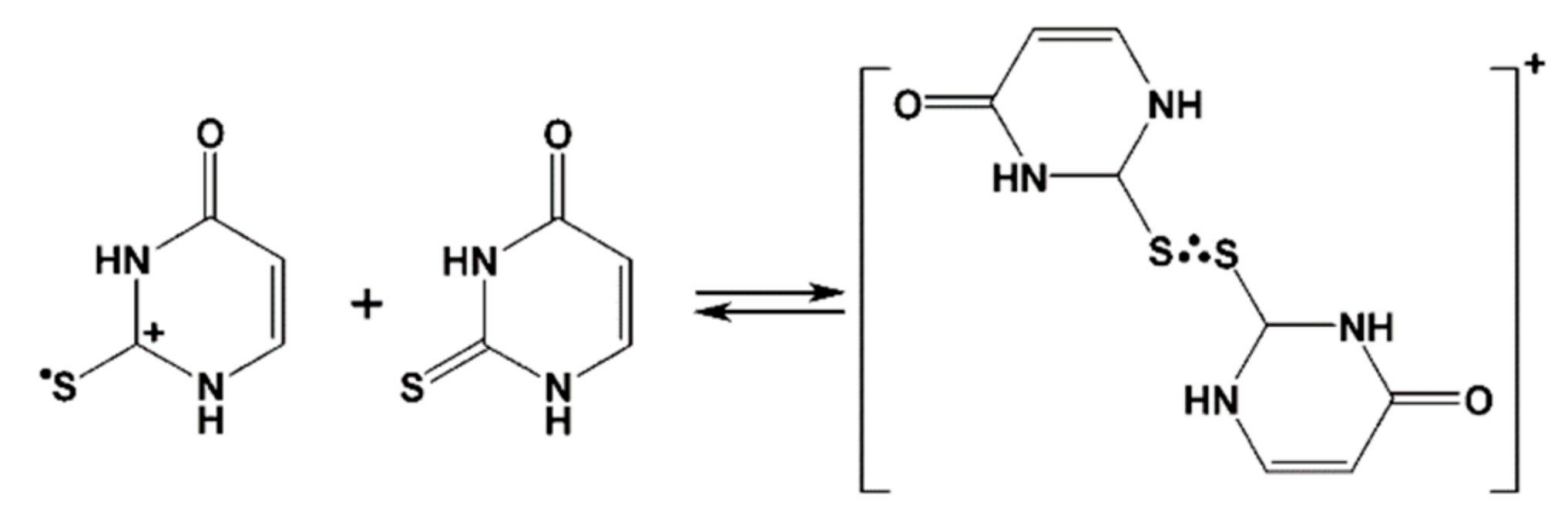
Dimeric radical cation formation in 2-thiouracil [58].

The main conclusions derived from the above mentioned radiation chemical studies is that substitution by sulfur substantially changes pathway of radical reactions. Sulfur atom is the main target of one-electron oxidation by ^•^OH, ${\mathrm{Br}}_{2}^{●-}$, ${\mathrm{Cl}}_{2}^{●-}$, leading to the formation of sulfur centered radical cation/thiyl radical, which instantaneously dimerize with excess of sulfur nucleobase, forming three-electron bonded, sulfur centered radical cation (see Scheme 1 for 2-TU) [58,59]).

On the other hand, in the presence of sulfur atom at the position C2 in 2-TU and 2-thiocytosine, one-electron reduction by $e_{\mathrm{aq}}^{-}$ leads only to radicals protonated on heteroatoms—an exocyclic O-atom in 2-TU and a ring N-atom in 2-thiocytosine [54].

**Scheme 2:**
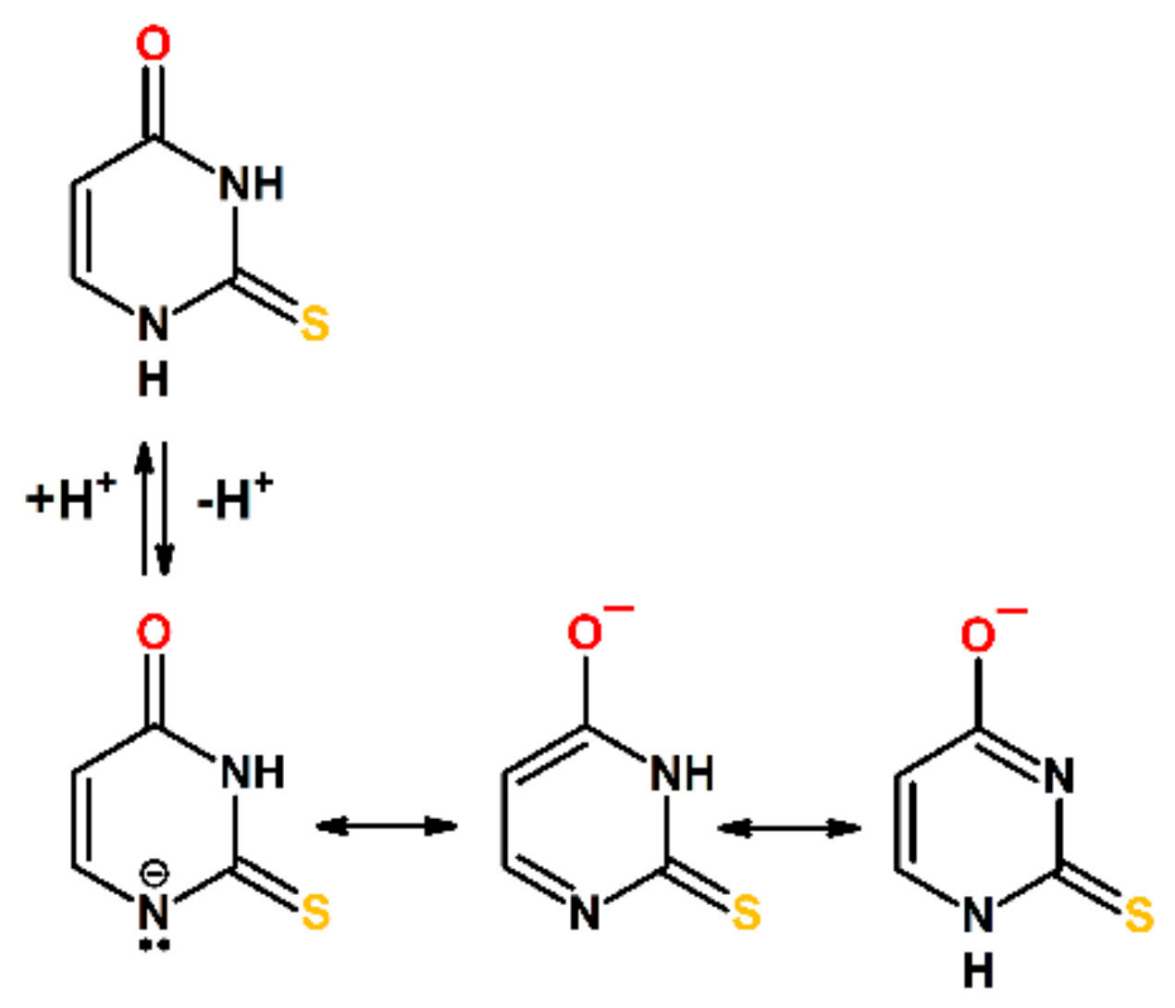
Acid-base and tautomeric equilibria in 2-TU.

Studies conducted so far have undoubtedly demonstrated a very complex mechanistic scenario of oxidation and reduction of sulfur substituted nucleobases. Moreover, the presence of a sulfur atom (instead of an oxygen atom) at position C2 in 2-TU may result in the establishment of an additional tautomeric equilibrium (in relation to uracil), namely between the thione and thiol form [60,61]. This is a key issue since the presence or lack of specific functional groups may drastically affect the reaction pathway. Theoretical [62,63,64] and experimental [65,66] studies revealed that the energetically preferred tautomer of the neutral form of 2-TU in solution is the oxo-thione (Scheme 2). On the other hand, it was also suggested that 2-TU exists as the equilibrium mixture of oxo-thione and oxo-thiol forms; however, the population ratio of tautomers were not evaluated [67]. The calculated relative free energies indicated that two out of several possible thiol tautomers (hydroxy-thiol/oxo-thiol) are energetically higher by about 50–60 kJ mol^−1^ compared to the oxo-thione tautomer [62,63,64]. In turn, anionic form of 2-TU (pK_a_ = 7.75) [68] exists in equimolar mixture of two hydroxy-thione tautomers in the form of monoanions (Scheme 2) due to deprotonation either at N1 or N3 nitrogen atoms [65].

With this information and premises, we report in this paper our in-depth studies on the ^●^OH and ^●^N_3_ radicals induced oxidation of 2-TU in aqueous solution. The current paper is dedicated to extension of previous studies on oxidation of 2-TU [58], addressing the influence of 2-TU concentration, pH and the character of one-electron oxidant on the transient absorption spectra and the kinetics of transients formed. The experimental transient absorption spectra observed in irradiated aqueous solutions containing 2-TU will be compared with the respective spectra of radicals and radical ions calculated using TD-DFT methods.

## 2. Results

### 2.1. Oxidation by ^●^OH Radicals—Influence of 2-TU Concentration on Absorption Spectra

**Figure 1:**
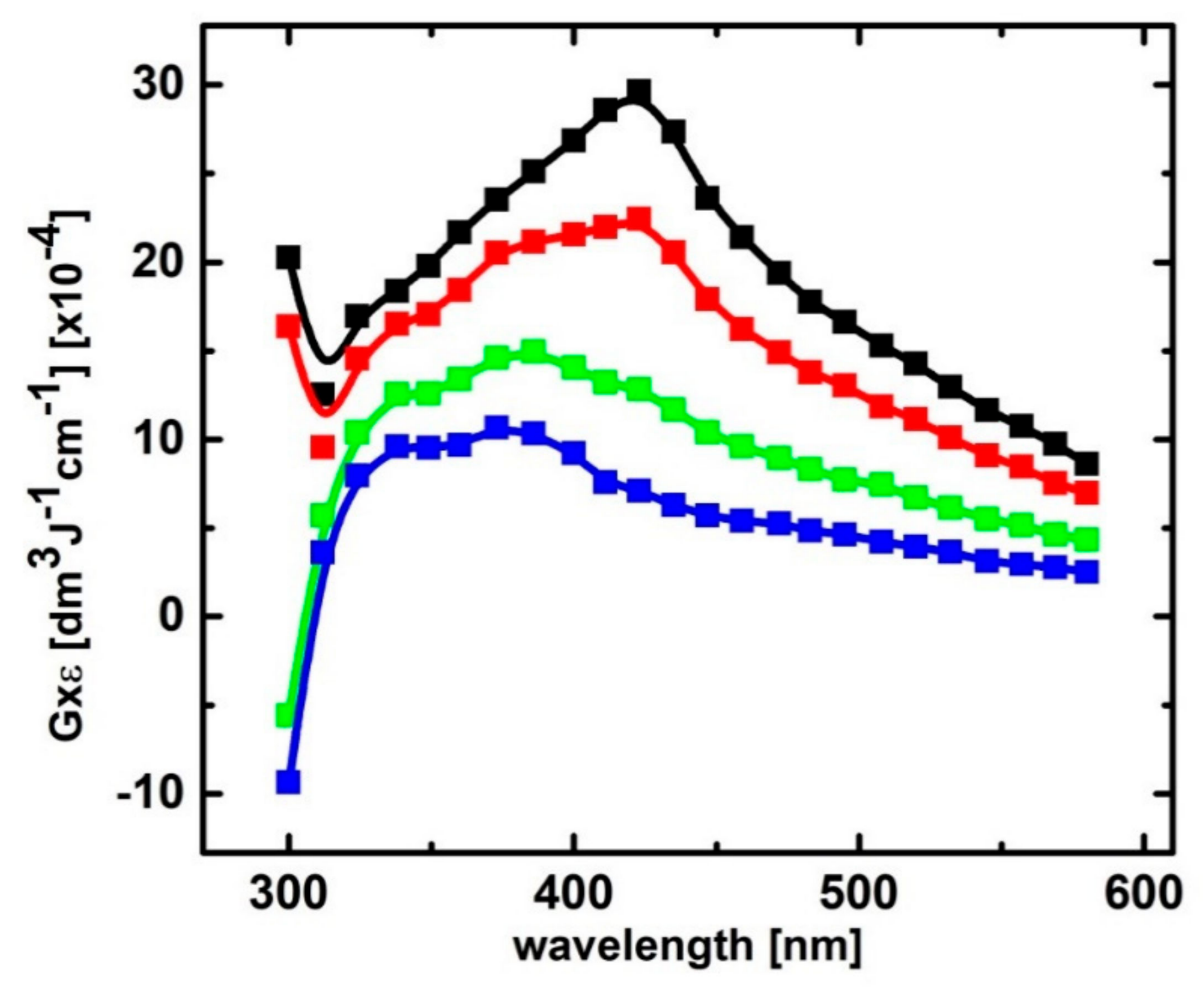
Transient absorption spectra recorded in N_2_O-saturated unbuffered aqueous solution at pH 4 containing 0.1 mM (■), 0.2 mM (■), 0.5 mM (■) and 1 mM (■) of 2-TU, recorded 3 μs, 2 μs, 1 μs and 0.8 μs, respectively, after electron pulse.

The transient absorption spectrum with λ_max_ = 430 nm, which resulted from the reaction of ^●^OH radicals with 2-TU present in aqueous solution in 1 mM concentration, reported in the earlier work [58], was assigned to the formation of dimeric radical cation with 2c-3e sulfur-sulfur bond (Scheme 1). It was the only intermediate identified as the oxidation product derived from 2-TU. Therefore, we decided to record UV-vis transient absorption spectra at various 2-TU concentrations in order to check whether and to what extent concentration of 2-TU affects spectral characteristics. The spectral changes obtained from the pulse irradiation of N_2_O-saturated unbuffered aqueous solutions containing 2-TU in the concentration range 0.1 mM to 1 mM are shown in Figure 1. The recording times are selected at the maximum intensity of the absorption signal after electron pulse for the specified concentration of 2-TU, which corresponds to the steady-state concentrations of transient species.

It is clearly seen that the absorption spectra recorded for two low concentration of 2-TU are different in shape and position of the absorption maxima in comparison to the absorption spectra recorded for two high concentration of 2-TU. They are characterized by a broad absorption band with a weakly pronounced maximum at λ ≈ 385 nm and a shoulder within 320–360 nm range. In turn, the latter two spectra are similar to the spectrum recorded in the earlier work and are characterized by λ_max_ ≈ 425 nm [58]. This is a strong indication that some other products are formed and their spectral contribution at higher 2-TU concentration is probably hidden under the absorption assigned earlier to the dimeric radical cation [58]. More experimental evidence for their formation are obtained by studying time evolution of absorption spectra at low 2-TU concentration.

#### 2.1.1. Time Evolution of Absorption Spectra—Low Concentration of 2-TU

**Figure 2:**
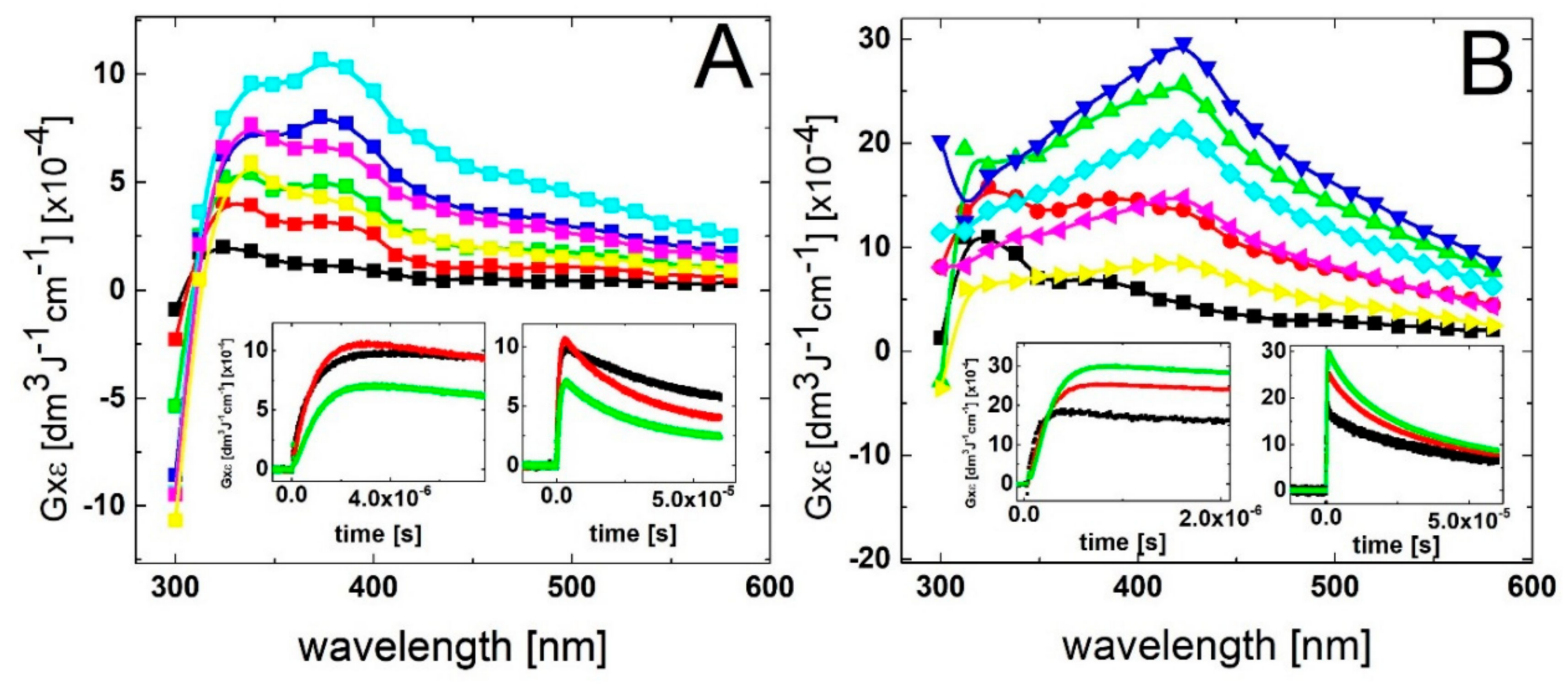
Transient absorption spectra recorded in N_2_O-saturated unbuffered aqueous solution at pH 4 containing (**A**) 0.1 mM of 2-TU 100 ns (■), 300 ns (■), 500 ns (■), 1 µs (■), 3 µs (■), 25 µs (■) and 60 µs (■) after electron pulse, (**B**) 1 mM of 2-TU 100 ns (■), 200 ns (■), 400 ns (■), 1 µs (■), 10 µs (■), 25 µs (■) and 60 µs (■) after electron pulse. Inserts: time profiles representing growth (left) and decay (right) of transient absorptions at λ = 338 nm (■), 386 nm (■) and 426 nm (■).

The spectral changes observed after pulse irradiation of N_2_O-saturated solutions containing 0.1 mM and 0.2 mM of 2-TU yielded complex series of spectral changes, however, their behavior in both cases is very similar (Figure 2A and Figure S1A in Supplementary Materials, respectively). A broad absorption spectrum with a weakly pronounced maximum at λ ≈ 325 nm and a shoulder within 360–385 nm range began to form within 300 ns time domain. With the time elapsed, both absorptions underwent further growth, however, after 1 μs with the inversion of intensities. The intensity of absorption at λ ≈ 385 nm became stronger and 2 μs (left insert in Figure S1A in Supplementary Materials) or 3 μs (left insert in Figure 2A) after the pulse the absorption spectra were fully developed and are characterized by a broad absorption band with λ_max_ ≈ 385 nm and a pronounced shoulder within 340–360 nm range.

With the time further elapsed, the absorption spectra observed at longer times (25 μs and 60 μs) after the pulse are characterized by a broad absorption bands, however, with the inversed position of λ_max_ (340 nm) and a shoulder (360–385 nm). Interestingly, no formation of the absorption band with a clear maximum at λ = 425 nm was observed at any time domain up to 60 μs, however, its presence this time might be probably hidden under the absorption band with λ_max_ = 385 nm. Nonetheless, the kinetic behavior followed at the two selected wavelengths (338 nm and 386 nm) indicates the presence of two transient species characterized by different lifetimes (right inserts in Figure 2A and Figure S1A in Supplementary Materials).

#### 2.1.2. Time Evolution of Absorption Spectra—High Concentration of 2-TU

The spectral changes observed after pulse irradiation of N_2_O-saturated solutions containing 0.5 mM and 1 mM of 2-TU yielded even more complex series of spectral changes in comparison to solutions with lower concentration of 2-TU. However, the time evolution of spectra observed within first 200 ns time domain is very similar. A broad absorption spectrum with weakly pronounced maxima at λ ≈ 325 nm and 385 nm was also observed (Figure S1B in Supplementary Materials and Figure 2B, respectively) as for low concentrations of 2-TU. However, with the time elapsed, the absorption spectra underwent further growth and also a substantial spectral shift. The absorption spectra were fully developed within 1 μs and are characterized by a broad absorption band with λ_max_ ≈ 425 nm (Figure S1B in Supplementary Materials and Figure 2B). Moreover, a pronounced shoulder within 360–385 nm is also seen for lower concentration of 2-TU (0.5 mM), (Figure S1B in Supplementary Materials).

With the time further elapsed, the absorption band with λ_max_ ≈ 425 nm started to decay and 10 μs after the pulse still dominated the spectra. However, the spectra observed at longer times (25 μs and 60 μs) after the pulse are characterized by a very broad and flat absorption without a clearly pronounced maximum (Figure S1B in Supplementary Materials and Figure 2B). The kinetic behavior followed at the three selected wavelengths (338 nm, 386 nm and 426 nm) (right inserts in Figure S1B in Supplementary Materials and Figure 2B) also indicates the presence of three different transient species.

#### 2.1.3. Influence of pH on Time Evolution of Absorption Spectra at Low and High Concentration of 2-TU

**Figure 3:**
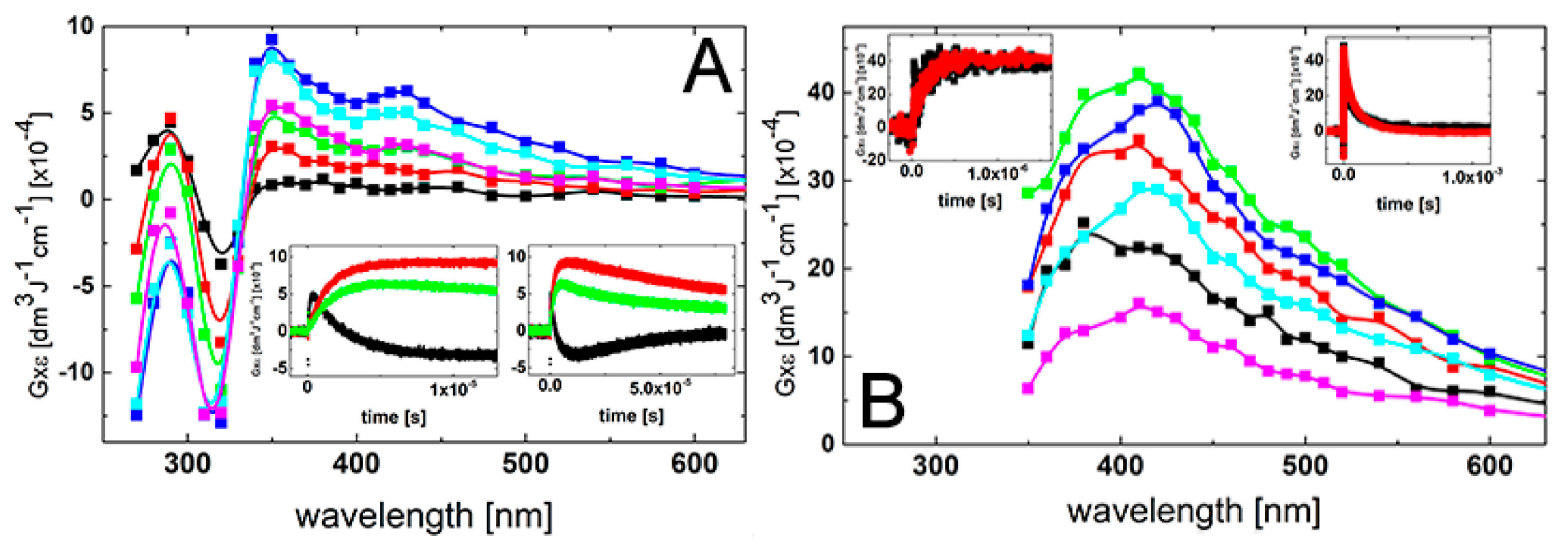
Transient absorption spectra recorded in N_2_O-saturated unbuffered aqueous solution at pH = 10 containing (**A**) 0.1 mM of 2-TU 200 ns (■), 500 ns (■), 1 μs (■), 6 μs (■), 20 μs (■), and 80 μs (■) after electron pulse. Inserts: time profiles representing growth (left) and decay (right) of transient absorptions at λ = 290 nm (■), 350 nm (■) and 420 nm (■); (**B**) 2 mM of 2-TU 100 ns (■), 240 ns (■), 1 μs (■), 6 μs (■), 25 μs (■), and 80 μs (■) after electron pulse. Inserts: time profiles representing growth (left) and decay (right) of transient absorptions at λ = 380 nm (■) and 420 nm (■).

The subsequent chemical systems subjected to irradiation were the basic aqueous solutions containing low (0.1 mM) and high (2 mM) concentration of 2-TU at pH = 10. At this pH, 2-TU exists in equimolar mixture of two hydroxy-thione tautomers in the form of monoanions (*vide* Scheme 2). In basic solutions, the spectral changes observed for the lowest concentration of 2-TU are different from those observed at pH = 4 as follows—(i) at short time domain up to 500 ns, a broad absorption spectrum with a weakly pronounced maximum at λ ≈ 350 nm (not at λ ≈ 325 nm) and a shoulder within 380–430 nm range (not within 360–385 nm range) began to form (Figure 3A). The first change in spectral features can be easily rationalized by higher absorption of 2-TU in the ground state < λ ≈ 350 nm at pH = 10 in comparison to pH = 4 (Figure S2 in Supplementary Materials) causing a stronger decrease in absorption due to the consumption of 2-TU. In turn, an appearance of a shoulder finds its justification in spectral features observed at 6 μs showing an absorption band with λ_max_ ≈ 430 nm. This absorption band was not observed at pH = 4 at low concentration of 2-TU (Figure 2A and Figure S1 in Supplementary Materials).

Meanwhile, the spectral changes observed for the highest concentration of 2-TU are very similar to those observed at pH = 4. At short time (100 ns), a broad absorption spectrum with a weakly pronounced maximum at λ ≈ 380 nm and a shoulder within 380–430 nm range was observed which with the further time elapsed is transformed into absorption spectrum with a clearly pronounced maximum at λ ≈ 425 nm. Moreover, due to the strong absorption of 2-TU in the ground state, measurements for λ < 350 nm were not possible (Figure S2 in Supporting Materials).

### 2.2. Oxidation by ^●^OH Radicals—Kinetics

#### 2.2.1. The Rate Constant of the ^●^OH Reaction with 2-TU

**Figure 4:**
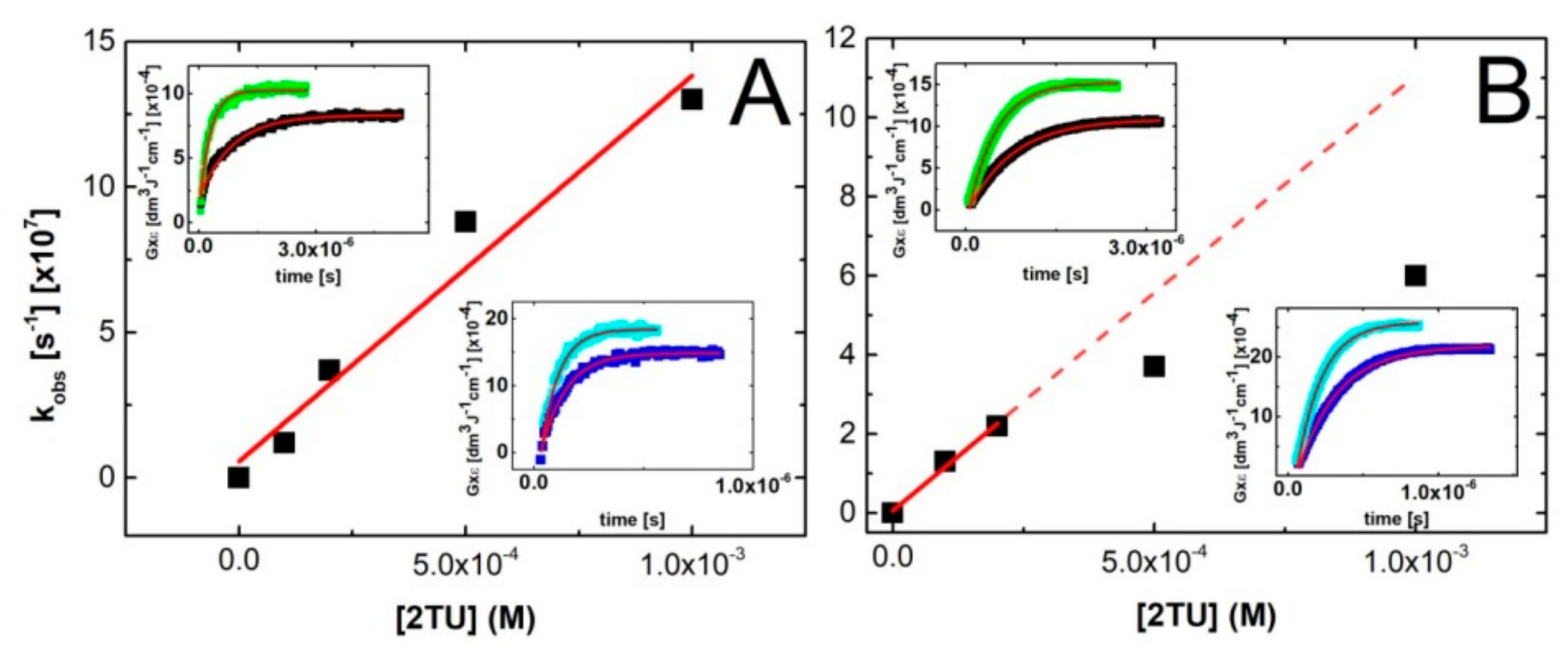
Plots of the observed pseudo-first-order rate constants of the formation of the 324-nm absorption (**A**) and the 386-nm absorption (**B**) as a function of 2-TU in N_2_O-saturated unbuffered aqueous solution at pH = 4. Inserts in A: time profiles representing growth of transient absorption at λ = 324 nm for the concentration of 2-TU (■) 0.1 mM and (■) 0.2 mM (top) and (■) 0.5 mM and (■) 1 mM (bottom). Inserts in B: time profiles representing growth of transient absorption at λ = 386 nm for the concentration of 2-TU (■) 0.1 mM and (■) 0.2 mM (top) and (■) 0.5 mM and (■) 1 mM (bottom).

In order to directly determine the bimolecular rate constant of the ^●^OH reaction with 2-TU, the build-up kinetics at various concentrations of 2-TU were recorded at two wavelengths—324 nm and 386 nm. The rate of formation, followed at these wavelengths fits to a single exponential (inserts in Figure 4). These wavelengths correspond to absorption maxima of two bands which were formed initially and were the only ones fully developed at low concentration of 2-TU (Figure 2A and Figure S1A in Supplementary Materials). These results support the tentative hypothesis that these absorption bands cannot be assigned to dimeric radical ions which are formed in a secondary process and are characterized by the absorption band with λ _max_ ≈ 425 nm [58]. With this information in hand, the pseudo-first-order rate constants of the formation of the 324-nm and 386-nm absorption were plotted as a function of 2-TU concentration (Figure 4). It is clearly seen that the pseudo-first-order rate constants measured at λ = 324 nm show a linear dependence on the concentration of 2-TU in the full range of concentration studied (Figure 4A) with the slope representing the second-order rate constant for the formation of transient(s) resulting from the reaction of ^●^OH radicals with 2-TU. The calculated second-order rate constant *k*_324_ = 1.3 × 10^10^ M^−1^s^−1^ is similar to the rate constants determined previously for 2-TU (9.6 × 10^9^ M^−1^s^−1^) [58] and thiourea (1.2 × 10^10^ M^−1^s^−1^) [69] by competition kinetics using 2-propanol.

On the other hand, the pseudo-first-order rate constants measured at λ = 386 nm show a departure from linearity for high concentration of 2-TU (Figure 4B). This is not surprising taking into account the following facts—high efficiency of dimeric radical ions formation at high concentration of 2-TU and significant contribution of their absorption band at this wavelength. In order to suppress this inconvenience, the pseudo-first-order rate constants measured for two lowest concentration of 2-TU were taken into account in the linear fit. The calculated second-order rate constant k_386_ = 1.1 × 10^10^ M^−1^s^−1^ fits very well to the expected rate constant value [58].

#### 2.2.2. Equilibrium Constant and Rate Constants of Reactions Involved in Equilibrium

For both pHs, the maximum value of the 426-nm absorbance is dependent on the 2-TU concentration. When this is increased from 0.047 mM to 3 mM, *G* × ε increases from 6.2 × 10^−4^ dm^3^ J^−1^ cm^−1^ to 25.6 × 10^−4^ dm^3^ J^−1^ cm^−1^ and from 5.6 × 10^−4^ dm^3^ J^−1^ cm^−1^ to 20.2 × 10^−4^ dm^3^ J^−1^ cm^−1^ (values corrected for the loss by bimolecular decay) for pH = 4 and 10, respectively (Figure S3, panels C and D). This increase cannot be accounted for by the increase in *G*(^●^OH) due to the higher 2-TU concentration [70]. It rather points to the existence of an equilibrium situation presented in Scheme 1 where a dimeric radical ion is formed which is responsible for the strong absorption at λ_max_ ≈ 425 nm.

The equilibrium constant *K* = *k*_forward_/*k*_backward_ can be obtained from Equation (1), where *A*_0_ is the absorbance at λ = 426 nm in 2-TU solution of 3 mM and *A* is the absorbance at a given concentration of 2-TU.

*A*_0_/*A* − 1 = *K*^−1^ [2-TU]^−1^ (1)

**Figure 5:**
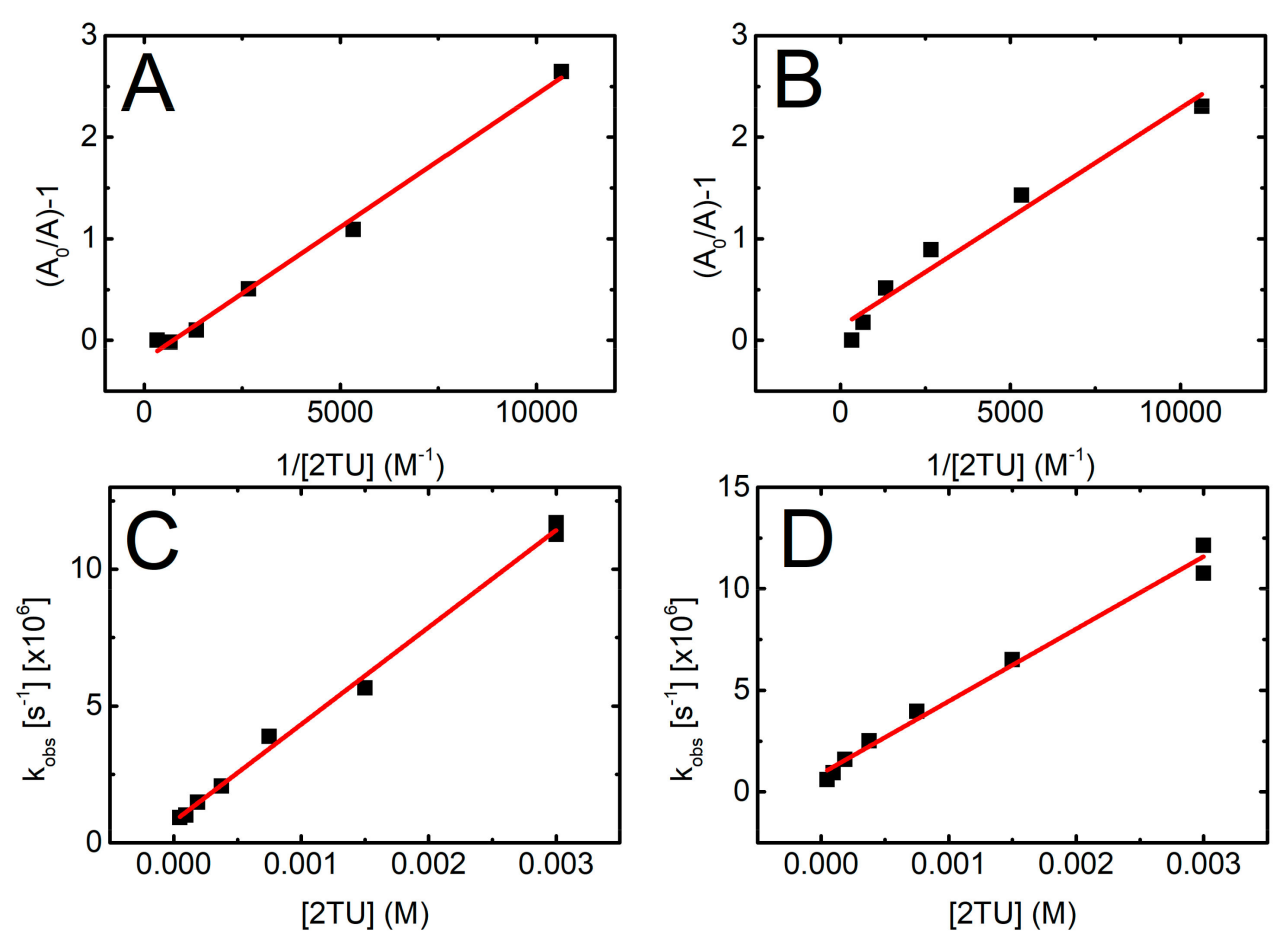
Dependence of the term (A_0_/A) − 1 at λ = 426 nm on the reciprocal of 2-TU in the pulse radiolysis of N_2_O-saturated unbuffered aqueous solution at pH = 4 (panel **A**) and pH 10 (panel **B**) containing 2-TU in the range of concentration 0.047 mm – 3 mM. Plots of the observed pseudo-first order rate constants of the formation of the 426-nm absorption as a function of 2-TU in N_2_O-saturated unbuffered aqueous solution at pH = 4 (panel **C**) and pH = 10 (panel **D**).

In Figure 5 (panels A and B), the term *A*_0_/*A* − 1 is plotted against the reciprocal of the 2-TU concentration for pH = 4 and pH = 10, respectively. From the reciprocal values of the slopes of these linear plots, K values 3.8(5) × 10^3^ M^−1^ and 4.6(5) × 10^3^ M^−1^ were obtained for pH = 4 and pH = 10, respectively. These values are very close to that reported for 2-TU at pH = 6.5 (4.7 × 10^3^ M^−1^) [58], however, substantially lower to that reported for 4-TU at pH = 7 (1.8 × 10^4^ M^−1^) [59] by using the same experimental approach.

A kinetic treatment of the equilibration process shown in Scheme 1 can also be represented by Equation (2), where *k*_obs_ is the experimental pseudo first-order rate constant for the formation of dimeric radical ion (Figure S3A,B in Supplementary Materials).

*k*_obs_ = *k*_forward_ [2-TU] + *k*_backward_ (2)

This approach allows measurement of not only equilibrium constant (*K*) but also rate constants involved in the equilibrium that is, (*k*_forward_ and *k*_backward_). Figure 5 (panels C and D) shows plots based on Equation (2) for pH = 4 and pH = 10, respectively. From the slopes and intercepts of the linear plots, we obtained *k*_forward_ = 3.6 × 10^9^ M^−1^s^−1^ and *k*_backward_ = 7.8 × 10^5^ s^−1^ for pH 4 and *k*_forward_ = 3.6 × 10^9^ M^−1^s^−1^ and *k*_backward_ = 9.0 × 10^5^ s^−1^ for pH = 10 and hence *K* = *k*_forward_/*k*_backward_ = 4.6 × 10^3^ M^−1^ and 4.0 × 10^3^ M^−1^ for pH = 4 and 10, respectively. Considering the errors in the measurement of absorbencies and rate constants based on relatively weak signals, the agreement in the respective *K* values obtained by the two approaches is very good. From these two independent measurements the average values for *K* are 4.2 × 10^3^ M^−1^ and 4.3 × 10^3^ M^−1^ for pH = 4 and pH = 10, respectively.

The obtained values of *k*_forward_ for 2-TU are comparable to that reported (5.0 × 10^9^ M^−1^s^−1^) in analogous equilibrium for thiourea. On the other hand, the obtained values of *k*_backward_ for 2-TU are significantly higher to that reported (9.1 × 10^3^ s^−1^) for thiourea [69]. These facts explain lower *K* values for 2-TU and indicates lower stability of its dimeric radical ion in comparison with thiourea.

### 2.3. Oxidation by ^●^OH Radicals—Time-Resolved Conductivity

It has been reported in a number of pulse radiolytic studies that time-resolved conductivity detection can help untangle mechanistic nuances encountered in analysis of kinetic changes of optical absorption especially when either multiple transients are formed in the similar spectral range [71,72] or the transients have optical absorption outside the available detection range [73]. Upon one-electron ^●^OH radical induced oxidation, hydroxide anion (OH^−^) is produced and instantaneously changes pH of irradiated solutions which manifests itself in change of apparent conductivity. In acidic conditions it results in the decrease of apparent conductivity as a consequence of neutralization reaction with protons (H^+^ + OH^−^→ H_2_O). In turn, in basic solutions it causes increase of apparent conductivity due to increase of concentration of conducting OH^−^ anions. From the amplitude of conductivity change the yield of ^●^OH radicals involved in one electron oxidation process can be easily deducted.

**Scheme 3:**
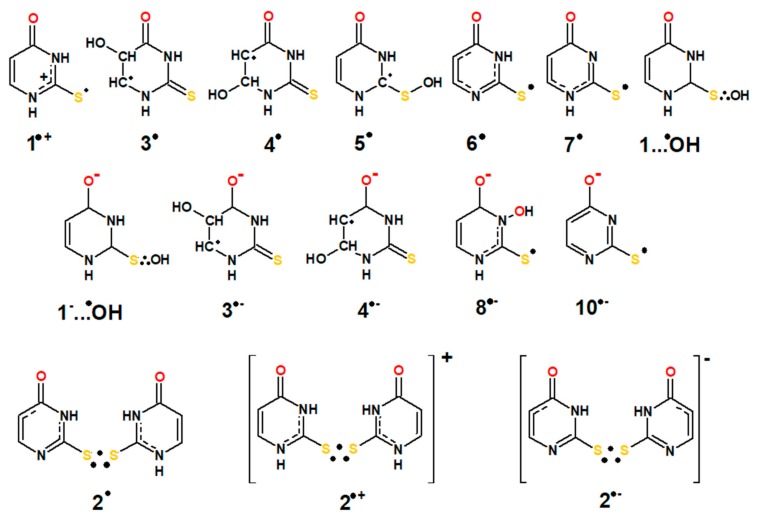
Structures of potential transients produced in the reaction of ^●^OH radicals with 2-TU at various pHs. The same symbols of transients were used as in the previous work [58] and expanded for the species which were not considered earlier.

In both acidic and basic N_2_O-saturated solutions of thiourea, a simpler compound with the same S=C < moiety like in 2-TU, transient conductivity changes after electron pulse were assigned to 100% yield of one-electron oxidation of thiourea to thiourea dimeric sulfur radical cation by ^●^OH radicals [69]. Since analogous dimeric radical cation formation was invoked in the previous pulse radiolytic studies of 2-TU [58], we wanted to estimate what extent of one-electron oxidation process, that is, the part of ^●^OH radicals yield, is involved in formation of radical cation 1^●+^, a potential precursor of dimeric radical cation 2^●+^ (Scheme 3).

**Figure 6:**
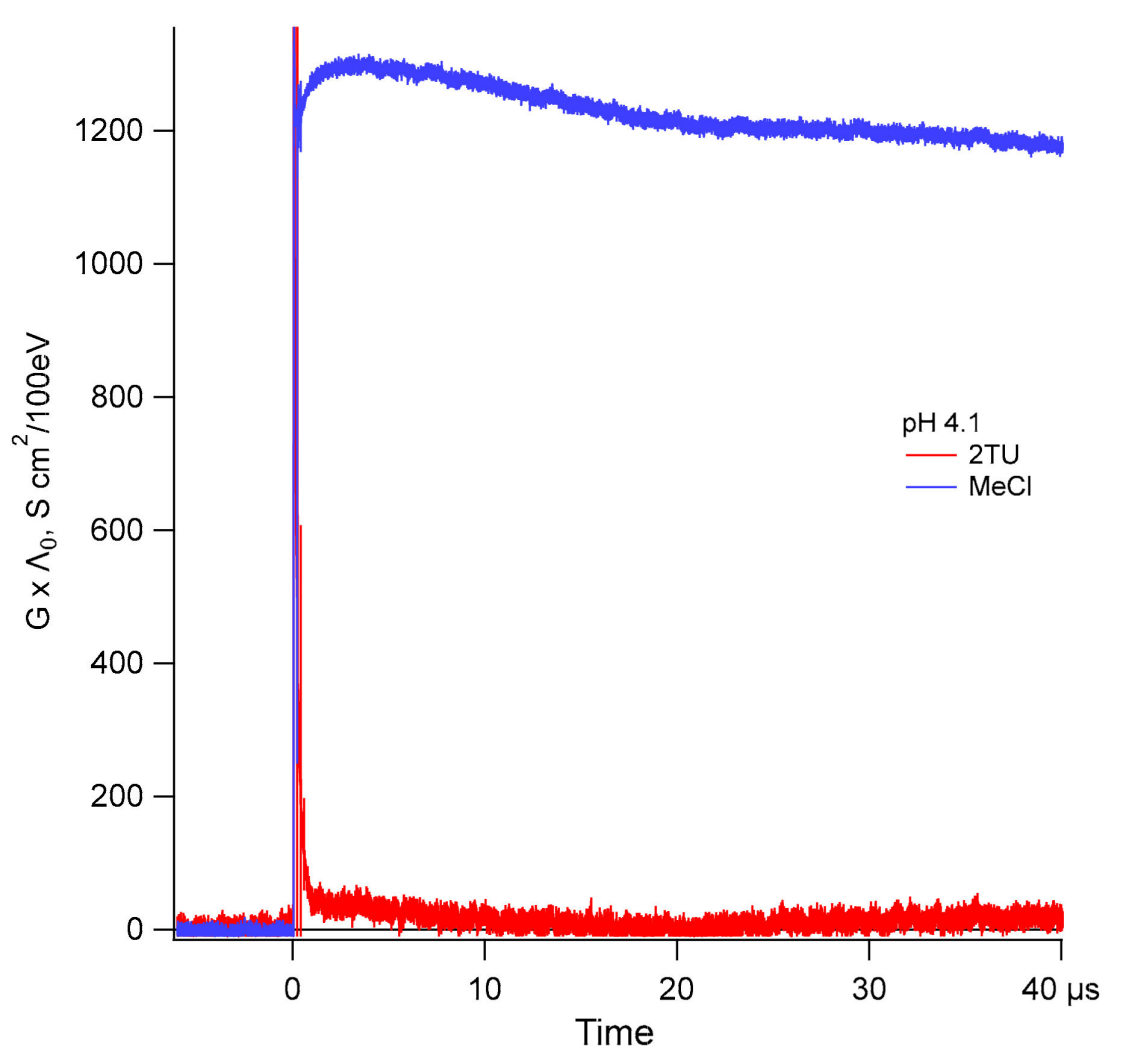
Comparison of equivalent transient conductivity changes represented as *G* × Λ_0_ vs. time after the pulse of electrons in N_2_O-saturated solution containing 1 mM of 2-TU at pH = 4.1 (red line) to CH_3_Cl-saturated aqueous solutions at the same pH (blue line).

Results of our studies are shown on Figure 6. After electron pulse in N_2_O-saturated 1 mM 2-TU acidic solutions (pH = 4.1) we observed an instantaneous growth of conductivity followed by its fast decrease almost to the level detected prior to the pulse (Figure 6, red line). The initial transient conductivity spike is a result of net increase of conductivity due to production of conducting species of water radiolysis (hydrated electrons ($e_{\mathrm{aq}}^{-}$) and protons (H^+^). In N_2_O-saturated aqueous solutions $e_{\mathrm{aq}}^{-}$ are quickly converted into ^●^OH radicals with the side product of hydroxide anions (OH^−^) being released as well within just few nanoseconds after the electron pulse. Therefore, the fast decrease of conductivity recorded within 1 µs after the pulse occurs through stoichiometric neutralization reaction (H^+^ + OH^−^ → H_2_O) with k = 1.4 × 10^11^ M^−1^s^−1^ [74], of highly conducting protons by OH^−^. Upon completion of this reaction stable conductivity level is reached and remains unchanged at the level of ~30 ± 10 S cm^2^/100eV for the next 80 microseconds (Figure 6 shows just first 40 µs).

### 2.4. Oxidation by ^●^N_3_ Radicals and CH_3_CN^●+^ Radical Cations

**Figure 7:**
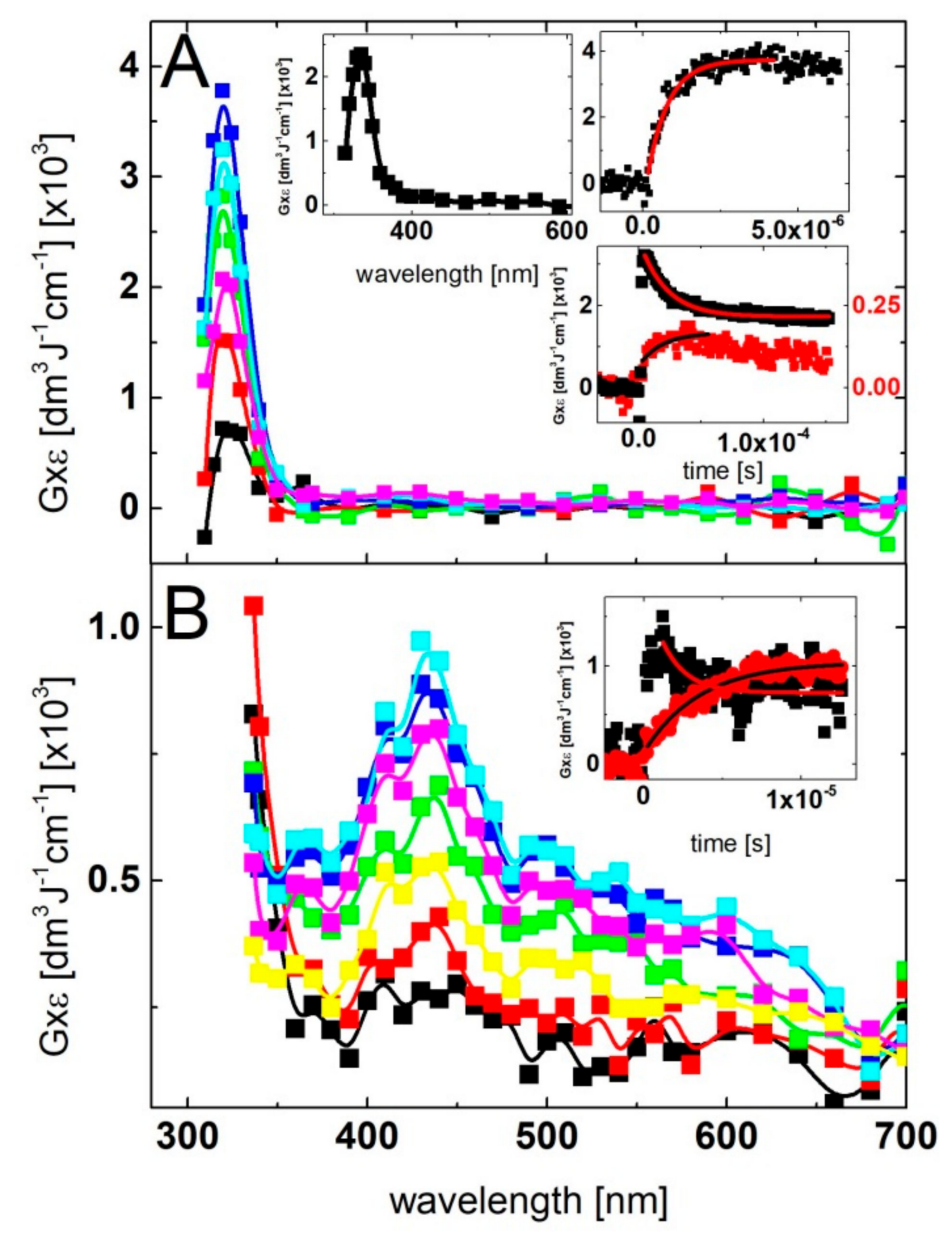
Transient absorption spectra recorded in N_2_O-saturated unbuffered aqueous solution at pH = 7 containing 50 mM NaN_3_ and (**A**) 0.1 mM of 2-TU 240 ns (■), 480 ns (■), 1 μs (■), 4 µs (■), 12 µs (■) and 60 µs (■) after electron pulse. Inserts: top left: transient absorption spectrum recorded 1 μs after the pulse in O_2_-saturated solution of acetonitrile containing 0.1 mM of 2-TU; top right: time profile representing growth of transient absorption at λ = 320 nm; bottom: time profiles representing growth of transient absorption at λ = 420 nm (■) and decay of transient absorption at λ = 320 nm (■); (**B**) 2 mM of 2-TU 600 ns (■), 1.2 μs (■), 3.2 μs (■), 8 µs (■), 12 µs (■), 24 μs (■) and 48 µs (■) after electron pulse. Inset: time profiles representing growth of transient absorption at λ = 420 nm (■) and decay of transient absorption at λ = 337nm (■).

The subsequent chemical systems subjected to irradiation were the N_2_O-saturated aqueous solutions containing NaN_3_ and two various concentrations of 2-TU (0.1 mM and 2 mM) at pH = 7. Taking into account the fact that oxidation potential of 2-TU at pH = 7 measured versus Ag/AgCl electrode falls in the vicinity of +0.5 V which is equivalent to ≈ +0.7 V versus SHE (the standard hydrogen electrode) [75] and the reduction potential of azide radicals (^●^N_3_) (+1.33 V vs. SHE) [76], one would reasonably expect that in such designed systems, oxidation of 2-TU can be initiated by ^●^N_3_ radicals. This was, indeed, observed. A transient absorption spectrum recorded 4 μs after pulse irradiation of N_2_O-saturated solutions containing 0.1 mM of 2-TU and 50 mM NaN_3_ exhibits a narrow and distinct absorption band with λ_max_ = 320 nm (Figure 7A) which is developed with *k* = 1.5 × 10^6^ s^−1^ (top right insert, Figure 7A). One has to note that similar absorption band was observed in O_2_-saturated acetonitrile solution containing 0.1 mM of 2-TU, where strongly oxidizing radical cations (CH_3_CN^●+^) are formed [77] and can also initiate oxidation of 2-TU (left top insert, Figure 7A). With the time further elapsed, the absorption spectra observed 12 μs and 60 μs after the pulse are still characterized by a similar 320-nm absorption band (Figure 7A). However, the decay kinetics of the 320-nm absorption band reached a plateau within 150 μs with *k*_320_ = 4.7 × 10^4^ s^−1^ (right bottom insert, Figure 7A). Interestingly, a kinetic trace recorded at λ = 420 nm represents a growth with *k*_420_ = 4.7 × 10^4^ s^−1^ (right bottom insert, Figure 7A). Comparison of the pseudo-first order rate constants measured at these two wavelengths clearly indicates that formation of the 420-nm absorption occurs at the expense of the decay of the 320-nm absorption.

The spectral changes observed after pulse irradiation of N_2_O-saturated solutions containing 2 mM of 2-TU are more visible in comparison to solutions with lower concentration of 2-TU. A transient absorption spectrum recorded 1.2 μs after the pulse is characterized by a strong nondescript absorption band with no distinct λ_max_ at wavelengths in the range 337-400 nm and a weaker, however pronounced, absorption band with λ_max_ ≈ 430 nm (Figure 7B). Due to the strong absorption of 2-TU in the ground state, measurements for λ < 337 nm were not possible (*vide* Figure S2 in Supporting Materials). The intensity of this uncharacteristic absorption band decreased in intensity (as shown by a decay of absorption measured at λ = 337 nm) and reached a plateau within 10 μs with *k*_337_ = 1.5 × 10^5^ s^−1^. At the same time absorption band with *λ*_max_ ≈ 430 nm was fully developed with *k*_420_ = 1.4 × 10^5^ s^−1^ (insert in Figure 7B). Comparison of the pseudo-first order rate constants measured at these two wavelengths clearly indicates that formation of the 420-nm absorption occurs at the expense of the decay of the 337-nm absorption. Excellent agreement in *k*_337_ and *k*_420_ values is a strong support that the species absorbing in the region < 340 nm is a direct precursor of the species absorbing at λ = 420 nm.

### 2.5. Theoretical Calculations

We expanded previous computational work on transient products formed in ^●^OH reaction with neutral form of 2-TU in aqueous solutions [58] to intermediates which can be produced with singly deprotonated 2-TU. The former should be observable in acidic solutions at pH = 4, the latter in basic solutions at pH = 10 (*vide* Scheme 2). The main intention of current work was to confront TD-DFT computations of potential transients (*vide* Scheme 3) with the experimental transient absorption spectra recorded at various concentrations of neutral and deprotonated 2-TU (*vide* Figure 2, Figure 3 and Figure S1 in Supplementary Materials). Upon encounter of ^●^OH with neutral or deprotonated 2-TU one-electron oxidation, ^●^OH addition (at positions C5, C6, S8) and H abstraction (at positions N1 and N3) can occur simultaneously. In order to help narrow down to the most efficient channels of ^●^OH-induced reactivity we performed detailed analysis of activation barriers of ^●^OH addition and H abstraction reactions.

Earlier work suggested [58] that transients produced with the highest yields have 2c-3e character. Therefore, we applied range separated hybrid functional ωB97x/aug-cc-pVTZ based computational methodology (*vide* Section 4.4) which proven quite successful in structural, spectral and thermochemical characterization of this group of open shell species [78,79]. Detailed solution phase optimized geometries of all transients with bond lengths as well as maximum spin populations are presented in Figures S4–S6 (Supplementary Materials). Our solution phase optimized geometries of monomer type transients (1^●+^, 1…^●^OH_(1)_, 1…^●^OH_(2)_, 3^●^, 4^●^, 6^●^, 7^●^) compare very well with the solution phase optimized geometries obtained previously at BH and HLYP/6-311+G(d,p) level [58].

Interestingly, the intermediates denoted as 1…^●^OH_(1)_, 1…^●^OH_(2)_ were found to be hemibonded ^●^OH adducts to sulfur atom. Typical shape expected for σ* SOMO orbitals in these type of intermediates is presented on Figure S7 (Supplementary Materials).

**Figure 8:**
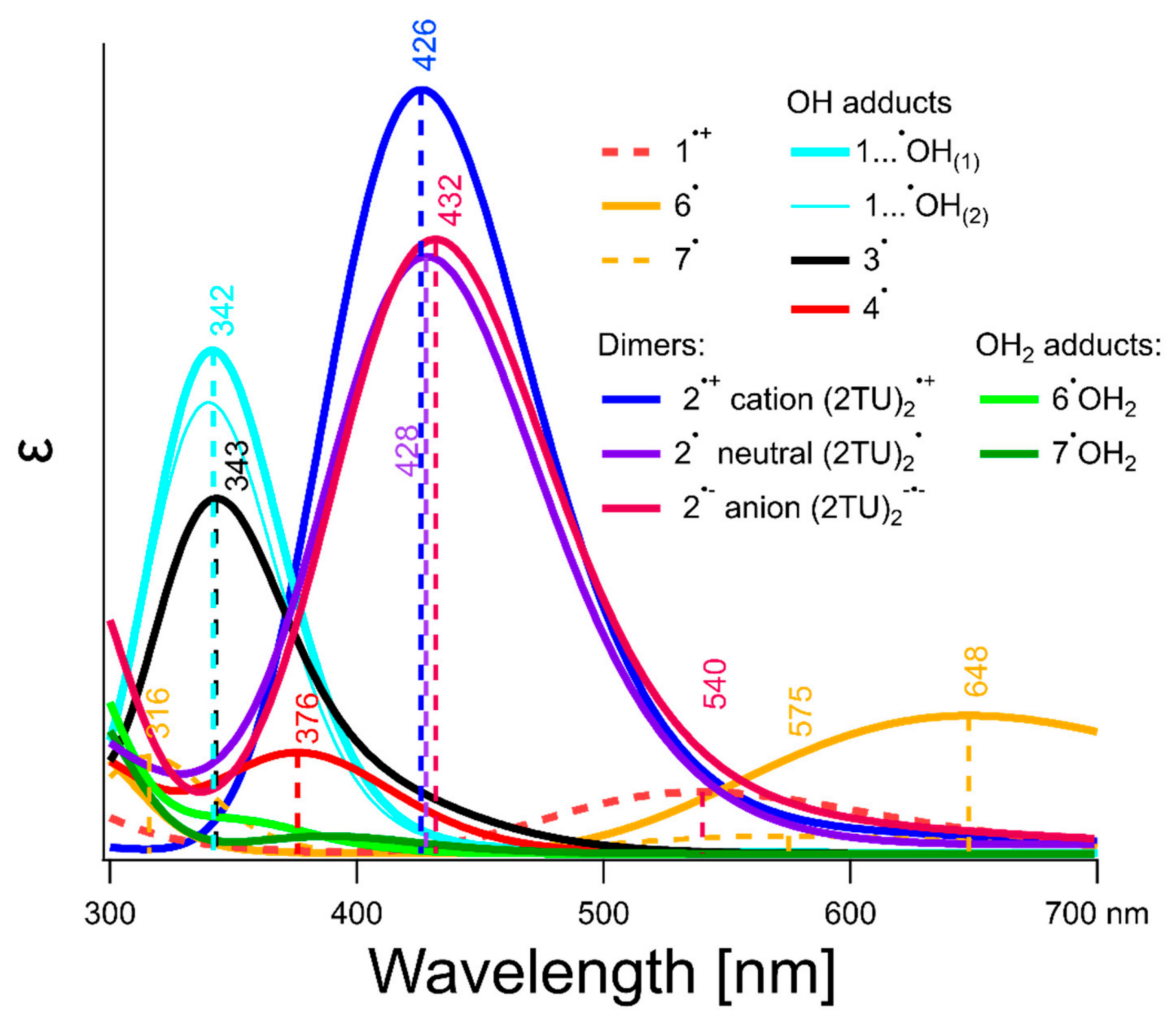
TD-DFT calculated absorption spectra of potential transients (see legend for symbols and Figures S4–S6 for geometries) produced in ^●^OH–induced oxidation of 2-TU in water at pH = 4.

The computed harmonic stretch frequencies of SO bonds in 2c-3e OH adducts are 329 cm^−1^ and 338 cm^−1^, respectively. These computed values match almost exactly the experimental stretching frequency of 338 cm^−1^ recorded for 2c-3e SO bond in DMS-OH radical by the time-resolved Raman spectroscopy [80] assuring further our assignment. It has to be noted that 1…^●^OH_(1)_, 1…^●^OH_(2)_ were suggested as possible precursors of radicals 6^●^ and 7^●^ in the process of direct H-atom abstraction from positions N1 and N3, respectively [58]. However, they were not considered as hemibonded ^●^OH adducts to sulfur atom (*vide* Scheme 3) but as hydrogen-bonded adducts to H7 and H9 at N1 and N3 atoms in the ring, respectively. We performed detailed studies of energy profiles for H atom abstraction from positions N1 and N3 in aqueous phase. Results of these studies are summarized on Figures S8, S9A and Table S2 in Supplementary Materials. Contrary to the previous work we found that both channels of H atom abstraction at positions N1 and N3 with transition states (TS6^●^ and TS7^●^), geometrically close to 1…^●^OH_(1)_ and 1…^●^OH_(2)_, are essentially barrierless in aqueous phase (Figure S8 in Supplementary Materials). There is a small 0.25 kcal mol^−1^ barrier in TS7^●^ formation (Figure S8B in Supplementary Materials) which should contribute to some regioselectivity of H atom abstraction at position N1. The products resulting from H atom abstraction at positions N1 and N3 are hemibonded radicals with 2c-3e bonds between sulfur and oxygen of water molecules produced out of original OH moieties of respective hemibonded OH adducts upon H addition to oxygen (denoted as 6^●^OH_2_ and 7^●^OH_2_ (*vide* Figure S7 in Supplementary Materials), illustrating shapes of their σ* orbitals). These S-O hemibonded intermediates are energetically about 6–7 kcal mol^−1^ above free energy of hydrated thiyl radicals. Hence, one can only assume that at room temperature these intermediates will be in equilibrium with radicals 6^●^ and 7^●^ (Figure S8 in Supplementary Materials). We believe that these two channels represent paths of proton coupled electron transfer (PCET) to oxygens of 2c-3e adducts 1…^●^OH_(1)_, 1…^●^OH_(2)_ in production of thiyl radicals 6^●^ and 7^●^. We also found the second H abstraction pathway at position N3 with 14.6 kcal mol^−1^ barrier at transition state TS7_2_^●^ and pre-reactive complex RC7^●^ in which ^●^OH radical is hydrogen bonded to oxygen atom of 2-TU. This channel even though kinetically not feasible also leads to thiyl radical 7^●^ (vide Figure S9A in Supplementary Materials). The group of hemibonded ^●^OH adducts to sulfur were characterized experimentally by optical absorption band with λ_max_ in the range of 330 nm–390 nm [81,82,83]. Hence, we can expect that 1…^●^OH_(1)_ and 1…^●^OH_(2)_ should have similar spectral features. However, in our experiment they may be too short lived to be observed due to the mentioned above conversion to SO hemibonded water radicals (6^●^OH_2_ and 7^●^OH_2_) in PCET process. These kind of water hemibonded intermediates have been characterized in the past to have characteristic optical absorption band due to charge transfer from solvent transition [84,85]. Even though their lifetime can be short, they can be populated in reaction with neighboring water molecules and be constantly reformed due to large abundance of neighboring water molecules. Therefore, we decided to compute their absorption spectra as well (Figure 8).

Contrary to the previous studies [58], we found that ^●^OH radical addition to the most nucleophilic C5 and C6 sites of 2-TU ring and formation of the respective radicals 3^●^ and 4^●^ have energetic barriers (*vide* Figure S9B and Table S2 in Supplementary Materials). Even though the most stable conformer produced in the course of these reactions, radical 4^●^ (radical with H atom pointing inward ring, *vide* Figure S4 in Supplementary Materials), is thermodynamically more stable than the most stable conformer of 3^●^ (equatorial conformer with H atom of OH group pointing outward ring, *vide* Figure S4 in Supplementary Materials) by almost 1.9 kcal mol^−1^. In spite of the fact that it is even more stable than the most stable ^●^OH adduct to sulfur by almost 13 kcal mol^−1^, chances of its formation are very small. Formation of the most thermodynamically stable conformers of ^●^OH adducts to 2-TU ring proceed through 3.5 and 5.6 kcal mol^−1^ barriers at C5 and C6, respectively (*vide* Figure S9B and Table S2 in Supplementary Materials). As a result, analogously to reactions of ^●^OH addition to uracil ring [86,87] one can expect regioselectivity in addition to position C5 over C6 but still kinetically formation of 2c-3e OH adducts to sulfur should dominate based on computed energy profiles for ^●^OH addition reactions to neutral 2-TU.

Our calculated UV-vis spectra monomer type transients derived from 2-TU (2TU^●+^, 2TU^●^ and ^●^OH adducts, hemibonded water adducts and thiyl radicals) are shown in Figure 8. Figure 8 shows only computed spectra of intermediates from 300 nm up since it was not possible to reliably measure anything < 300 nm. Aqueous solutions of 2-TU attenuate analyzing light in that region due to its light absorption (Figure S2 in Supplementary Materials). This causes mixing of bleaching and absorbance signals which cannot be resolved without knowledge of individual contributions of all possible intermediates.

Among all monomer type transients derived from 2-TU expected to be produced at pH = 4, the strongest absorbing species are 1…^●^OH_(1)_, 1…^●^OH_(2)_ with their respective σ → σ* electronic transitions located near 342 nm–343 nm. The ^●^OH adducts to the most nucleophilic C5 and C6 sites of 2-TU ring (radicals 3^●^ and 4^●^, respectively) absorb light in the similar spectral range according to our computations. Their absorption bands are characterized by absorption maxima located at λ = 343 nm and 376 nm, respectively (Figure 8).

In the process of geometry optimization of dimeric radical cation 2^•+^ we noticed that this intermediate can exist in three different rotamers produced by 180° torsional rotation around C─S bonds. All isomers should be observable since the energy difference between them is less than 0.5 kcal mol^−1^. The optimized geometry of the isomer of 2^•+^ with the lowest energy has the same structure as presented before [58] and is shown in Figure S6 (Supplementary Materials). All of the three isomers have almost identical absorption spectrum with λ_max_ located at 426 nm (*vide* 2^•+^ in Figure 8) and slightly varying oscillator strength owing to the fact that σ and σ* orbitals are orthogonal and apparently are not much affected by the slightly varying geometries of both pendant molecular rings. Considering that either radical cation 1^•+^ can deprotonate in position N1 giving more thermodynamically stable radical 6^•^ (rather than radical 7^•^ formed upon deprotonation in position N3) or H-atom abstraction by ^●^OH radicals can also preferably happen at position N_1_ (*vide supra* and Figure S8 in Supplementary Materials*)*, we can anticipate formation of neutral dimeric radical 2^•^ resulting from dimerization of radical 6^•^ with neutral 2-TU. Similar scenario can be expected upon dimerization of radical 7^•^ with neutral 2-TU. We considered both possibilities computationally and found that dimer resulting from dimerization of radical 6^•^ with neutral 2-TU is thermodynamically more stable. Stability of geometry of radical 2^•^ depends on internal hydrogen bonding between N atoms and H atoms of the nearest protonated nitrogen. Rotation of the fully protonated 2-TU around C─S bond by 180° leads to its isomeric form. Two most stable isomers differ less than 0.7 kcal mol^−1^ in their respective energies. Optimized geometry of the most stable isomer of radical 2^•^ is presented on Figure S6 (Supplementary Material). Calculated UV−Vis spectrum of this form of radical 2^•^ is shown in Figure 8. Optical spectral parameters of radical 2^•^ differ slightly as far as position of λ_max_ is concerned and shows only 20% smaller oscillator strength. Isosurfaces of computed spin density of all 2c-3e intermediates considered in our studies are shown in Figure S7 (Supplementary Materials).

At pHs higher than the first pK_a_ of 2-TU pronounced formation of anionic form of 2c-3e SS radical anion 2^●^^−^ can be readily expected in reaction of radical 6^●^ with deprotonated 2-thiouracil (2-TU^−^) at N1 position. Calculated UV−Vis spectrum of 2^●^^−^ is shown on Figure 8 and Figure S11 (Supplementary Materials). Absorption maximum of the anion radical 2^●^^−^ is slightly red-shifted in comparison to absorption maxima of radicals 2^●^ and 2^●+^ and has smaller oscillator strength than dimeric radical cation 2^●+^ but a bit larger than neutral dimeric radical 2^●^.

Additional forms of deprotonated monomer-type transients (1_a_^−^…^●^OH_(1)_, 1_a_^−^…^●^OH_(2)_, 1_b_^−^…^●^OH_(1)_, 1_b_^−^…^●^OH_(2)_, 3_a_^●^^−^, 4_a_^●^^−^,3_b_^●^^−^, 4_b_^●^^−^, 8^●^^−^, 10^●^^−^) can be observed at pH = 10 due to ^●^OH reaction with singly deprotonated 2-TU (i.e., 2-TU_a_^-^ or 2-TU_b_^−^, where subscripts ‘a’ or ‘b’ indicates the site of deprotonation at N1 or N3, respectively). Their optimized geometries are presented on Figure S5 (Supplementary Materials).

The energy profiles of deprotonated monomer-type transients formation are quite similar to energy profiles of their protonated counterparts generated at pH ~ 4. ^●^OH addition to sulfur has no activation barrier and should dominate kinetically regioselectivity of all ^●^OH addition reactions (*vide* Figure S10 in Supplementary Materials). Hemibonded negatively charged OH adducts are geometrically very close to transition states leading to almost barrierless H atom abstraction from positions N1 and N3. Unlike acidic conditions H abstraction at N3 has now small energy barrier of 0.17 kcal mol^−1^. Either of two paths of a PCET in deprotonated 2-TU will lead first to a hemibonded SO water intermediate 10_a_^●^^−^OH_2_ or 10_b_^●^^−^OH_2_ (where ‘a’ or ‘b’ specifies deprotonation site in 2-TU molecule at N1 or N3, respectively) which then rearranges to thiyl radical anion 10^●^^−^. In turn this radical can react with hydrating water molecule giving back water hemibonded 10_a_^●^^−^OH_2_ or 10_b_^●^^−^OH_2_. An alternate route for N3 H atom abstraction, also resulting in thiyl radical anion 10^●^^−^, has a barrier of 13.6 kcal mol^−1^ and proceeds from pre-reaction complex RC10_a2_^●^^−^ which consists of ^●^OH radical H-bonded to oxygen atom of 2-TU_a_^−^ (Figure S10A in Supplementary Materials). It is worth noting that, unlike in neutral form, in singly deprotonated thiobase a PCET process can proceed only on one side of a sulfur atom, where a nitrogen atom is protonated. This means that the opposite side of the sulfur is free to form a 2c3e OH adduct, which should have a longer lifetime due to hydrogen bonding between OH and deprotonated nitrogen site (for structures see Figure S5 and for spin density isosurfaces Figure S7). Since 1_a_^−^…^●^OH_(2)_ and 1_b_^−^…^●^OH_(1)_ are involved in a PCET, it means that in solutions at lowest thiobase concentration 1_b_^−^…^●^OH_(2)_ and 1_a_^−^…^●^OH_(1)_ may be longer observable than hemibonded OH adducts produced in analogous acidic conditions. The presence of tautomeric forms of 2-TU becomes relevant at pHs couple units below first pK_a_ of thiobase and therefore one can expect spectral contribution of intermediates 1_b_^−^…^●^OH_(2)_ and 1_a_^−^…^●^OH_(1)_ already at nearly neutral pH in sub-millimolar thiobase solutions, where hemibonded SS dimers formation is inhibited.

Among all 2-TU anionic monomer-type intermediates the strongest absorbing species are again hemibonded OH adducts to sulfur atom (1_a_^−^…^●^OH_(1)_, 1_a_^−^…^●^OH_(2)_, 1_b_^−^…^●^OH_(1)_, 1_b_^−^…^●^OH_(2)_). Their absorption spectra are characterized by comparable intensities to their protonated counterparts observed at pH 4 (Figure 8), however, with λ_max_ locations shifted to longer wavelengths and varying in the range between 353 nm and 367 nm (Figure S11 in Supplementary Materials). Upon deprotonation of 2-TU also absorption spectra of anionic ^●^OH adducts to the most nucleophilic C5 and C6 sites of 2-TU ring (labeled as radicals 3_a_^●^^−^/3_b_^●^^−^ and 4_a_^●^^−^/4_b_^●^^−^, respectively) are different in comparison to their protonated forms. Their absorption maxima are varying in the range of λ_max_ between 314 nm and 477 nm, respectively (Figure S11 in Supplementary Materials).

Additionally, we considered formation of ^●^OH adduct at N3 position, since in basic solutions 2-TU can form double C=N bond. We labeled this ^●^OH adduct 8^●^^−^. However since its formation is endothermic by 24 kcal mol^−1^ and its absorption spectrum is located <300 nm, this intermediate is not relevant to our discussion (for structure, thermochemistry and geometry of all discussed intermediates see Supplementary Materials Figures S5/S6 and Tables S1 and S3, respectively).

## 3. Discussion

### 3.1. Assignment of the Absorption Spectra to Respective Intermediates

The first key issue which has to be clarified before an attempt to propose the mechanism of one-electron oxidation of 2-TU by ^●^OH and ^●^N_3_ radicals is an assignment of the experimental transient spectra observed at low and high concentration of 2-TU to appropriate intermediates based on theoretical calculations and possible specific reactions of ^●^OH and ^●^N_3_ radicals. Earlier studies on reactions of ^●^OH radicals and dihalogen radical anions (${\mathrm{Cl}}_{2}^{●-},\;{\mathrm{Br}}_{2}^{●-}$) with 2-TU reported the formation of only one intermediate, namely, dimeric radical cation (2-TU)^●+^ characterized by an absorption band with λ_max_ = 430 nm and which exists in an equilibrium shown on Scheme 2 [58]. However, except for theoretical calculations, there were no other proofs that its precursor and dimeric radical itself have cationic form. Moreover, no experimental transient spectra which can be assigned to other possible transients resulted from 2-TU oxidation were reported. One of the reason was that the earlier spectral studies were limited to only one and relatively high concentration of 2-TU [58].

*Low concentration of 2-TU*. Thus, what is the identity of the transient species absorbing in the wavelength range 320–400 nm and characterized by weakly pronounced absorption maxima located at λ_max_ = 325, 340 and 385 nm (*vide* Figure 2A and Figure S1A in Supplementary Materials) in solutions containing low concentration of 2-TU at pH = 4? Taking into account the molecular structure of the most energetically favorable tautomer of 2-TU (Scheme 2) and plausible primary reactions of ^●^OH radicals, it is reasonable to assume the following primary sites of ^●^OH-attack—(i) a double C=C bond at C5 and C6 position and (ii) a double C=S bond at S8 position. The ^●^OH adducts at C5 and C6 positions labelled as 3^●^ and 4^●^ (Scheme 3) are characterized by absorption bands with calculated maxima located at λ = 343 nm and 376 nm, respectively (Figure 8). By analogy to uracil, C5 position is of the higher electron density than C6 position and should be more preferred site to electrophilic ^●^OH attack [88]. Moreover, their relative free energies in solution (∆G_rel_), with respect to the sum of substrates (^●^OH and 2-TU) are lower (−6.85 kcal mol^−^^1^ and −10,53 kcal mol^−^^1^, respectively) and therefore their formation is thermodynamically possible [58]. Even though the most stable conformer produced in the course of these reactions, radical 4^●^, is thermodynamically more stable than the most stable conformer of radical 3^●^ (*vide* Figure S9B and Table S2 in Supplementary Materials) its formation proceeds through 5.6 kcal mol^−1^ barrier in comparison to 3.5 kcal mol^−1^ calculated for 3^●^. Therefore, we can expect higher contribution of 3^●^ in experimental absorption spectra presented in Figure 2A and Figure S1A in Supplementary Materials.

An interesting case is an addition of ^●^OH to S8 in a double C=S bond. This reaction can potentially lead to the C2-centered radical labelled as 5^●^ (Scheme 3) characterized by absorption band with calculated maximum at λ = 340 nm. However, its formation is thermodynamically unfavorable [58] and 5^●^ is not considered as a species contributing to experimental absorption spectra observed in this work. However, this kind of radical was considered as a primary species during oxidation of thiourea by ^●^OH radicals [69].

Numerous theoretical studies about the character of bond in ^●^OH radicals adducts to sulfur atom has been performed over the years and in organic sulfides, in particular, 2c-3e character of the S-O bond was established [89,90]. This fact motivated us to consider similar intermediate, namely ^●^OH adduct to sulfur atom in a double C=S bond, having 2c-3e bond character. The OH adducts at S8 position labelled as 1…^●^OH_(1)_, 1…^●^OH_(2)_ (Scheme 3) meet all requirements for hemibonded ^●^OH adducts to sulfur atom. The S−O bond length is ~2.33 Å and maximum spin population is almost evenly distributed over S and O atoms (*vide* Figure S4 in Supporting Materials). What is equally important both 1…^●^OH_(1)_, 1…^●^OH_(2)_ radicals are characterized by absorption bands with calculated maxima located at λ in the range 340–342 nm (Figure 8) and can also contribute to experimental absorption spectra presented in Figure 2A and Figure S1A in Supplementary Materials. However, one can reasonably assume that 1…^●^OH_(1)_, 1…^●^OH_(2)_ radicals are precursors of 6^●^ and 7^●^ radicals which can be formed in the concerted electron/proton transfer where formation of 1^●+^ occurs in concert with its deprotonation at positions N1 and N3, respectively. Alternatively, it would not be surprising, that the both hemibonded ^●^OH adducts to sulfur can be precursors of radical cation 1^●+^ (Scheme 3) via analogous spontaneous and/or proton catalyzed dissociation processes which were observed in ^●^OH-induced oxidation of aliphatic sulfides [91] and methionine containing peptides [71,72,92]. However, based only on changes of absorption spectra, it was not possible to determine which reaction pathway takes place. However, these reaction pathways can be distinguished based on net changes of the apparent conductivity (*vide* Section 3.2).

The absorption spectra recorded at pH = 10 (Figure 3A), taking into account the molecular structure of the most energetically favorable tautomers of 2-TU (Scheme 2), can be in principle assigned to the similar type of species as for pH = 4, that is, 1^−^…^●^OH_(1)_, 1^−^…^●^OH_(2)_, 3^●^^−^ and 4^●^^−^ (Scheme 3). The ^●^OH adducts at S8 position meet also all requirements for hemibonded ^●^OH adducts to sulfur atom. The S−O bond length is ~2.38 Å and maximum spin population is almost evenly distributed over S and O atoms (*vide* Figure S5 in Supplementary Materials). Interestingly, the absorption band with λ_max_ = 425 nm was also observed, which can be assigned to dimeric radical anion 2^●^^−^ based on calculated λ_max_ = 432 nm (Figure S11 in Supplementary Materials).

*High concentration of 2-TU*. The transient absorption spectra observed at high concentration of 2-TU are similar (Figure S1B in Supplementary Materials and Figure 2B). to that observed in the earlier work and were assigned to the formation of dimeric radical cation with 2c-3e sulfur-sulfur bond 2^●+^ [58]. As a consequence, monomeric radical cation 1^●+^ was taken as a direct precursor of 2^●+^ which is presented in the form of equilibrium (*vide* Scheme 1).

In our calculations, we considered 2^●+^ as a possible transient responsible for the absorption band with λ_max_ = 425 nm, however, we considered also possibility of formation of dimeric radicals in neutral (2^●^) and anionic (2^●^^−^) forms. Since their calculated spectral parameters are very similar to that calculated for 2^●+^ (Figure 8) their character and as well of their direct precursors cannot be decided on the basis of absorption spectra. The observed net change of apparent conductivity should dispel doubts about the character of the dimeric radical formed (*vide* Section 3.2).

There is no reason to exclude contribution of the ^●^OH adducts at C5 and C6 positions labelled as 3^●^ and 4^●^ formed at low concentration of 2-TU to the observed transient spectra. Their presence is clearly manifested in absorption spectra at short time domains (Figure 2B and Figure S1B in Supporting Materials), however, at long time domains are hidden under the absorption assigned to one of the dimeric radical forms.

### 3.2. Justification of the Reaction Pathway Involving Hemibonded ^●^OH Adducts to Sulfur Atom

Contrary to our expectations, based on previous results with thiourea [69], decrease of the net conductivity below zero was not observed which would imply consumption of H^+^ through neutralization reaction by OH^−^ (*vide* Section 2.3) which are released with simultaneous formation of radical cation 1^●+^, in another words replacement of highly conducting protons by the resulting low conducting radical cations 1^●+^. Hence, formation of 1^●+^ followed by formation of 2^●+^ does not seem to be the reaction pathway during ^●^OH induced oxidation of neutral form of 2-TU.

Instead, there is a couple of possible scenarios which can be suggested. The first one, in which ^●^OH radicals react mainly by processes of addition and H-atom abstraction upon which no new conducting species are being formed. This process was suggested earlier, however, with activation energies in the range 2.53 kcal mol^−1^–3.79 kcal mol^−1^ [58]. Taking also into account an additional well known fact that H-abstraction reactions by ^●^OH radicals occur with lower rate constants than their addition to sulfur atom, this possibility can be rather discarded. The second one seems to be more reasonable. The ^●^OH radicals form the hemibonded adduct to sulfur (1…^●^OH) which decays by separated coupled electron proton transfer. The OH^−^ generated in the inner-sphere electron transfer, (that leads simultaneously to radical 1^●+^) is neutralized by the proton released from either N1 or N3 atoms of 1^●+^ and therefore formation of 6^●^ or 7^●^ is not associated with any net change of conductivity. Moreover, the lack of decrease of the net conductivity below zero excludes the process which operates in case of methionine containing peptides [71,72,92]. In this processes, the hemibonded S∴OH radical undergoes OH^−^ elimination, however, by protons from the bulk of solution, leading simultaneously to monomeric radical cation (S^●+^) and further to dimeric radical cation with 2c-3e S─S bonds (S∴S)^+^. This process is accompanied by decrease of net conductivity due to substitution of highly conducting protons by the resulting low conducting radical cations S^●+^ and (S∴S)^+^. This observation is crucial in determining the nature of both the dimer radical and its precursor (*vide* Section 3.3).

### 3.3. Mechanisms of ^●^OH and ^●^N_3_-Induced Oxidation of 2-Thiouracil

**Scheme 4:**
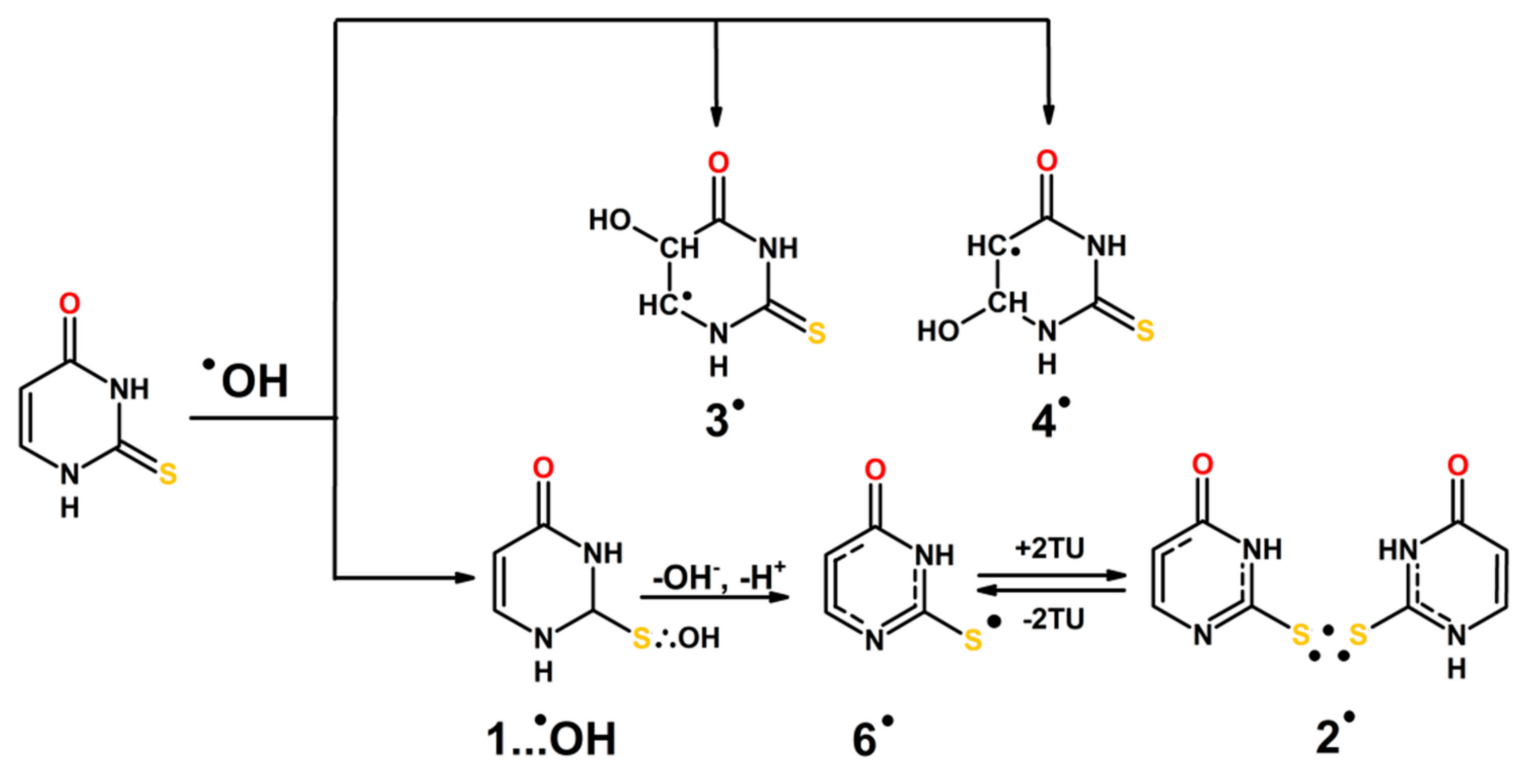
^●^OH-induced oxidation of 2-TU at pH below its pK_a._

**Scheme 5:**
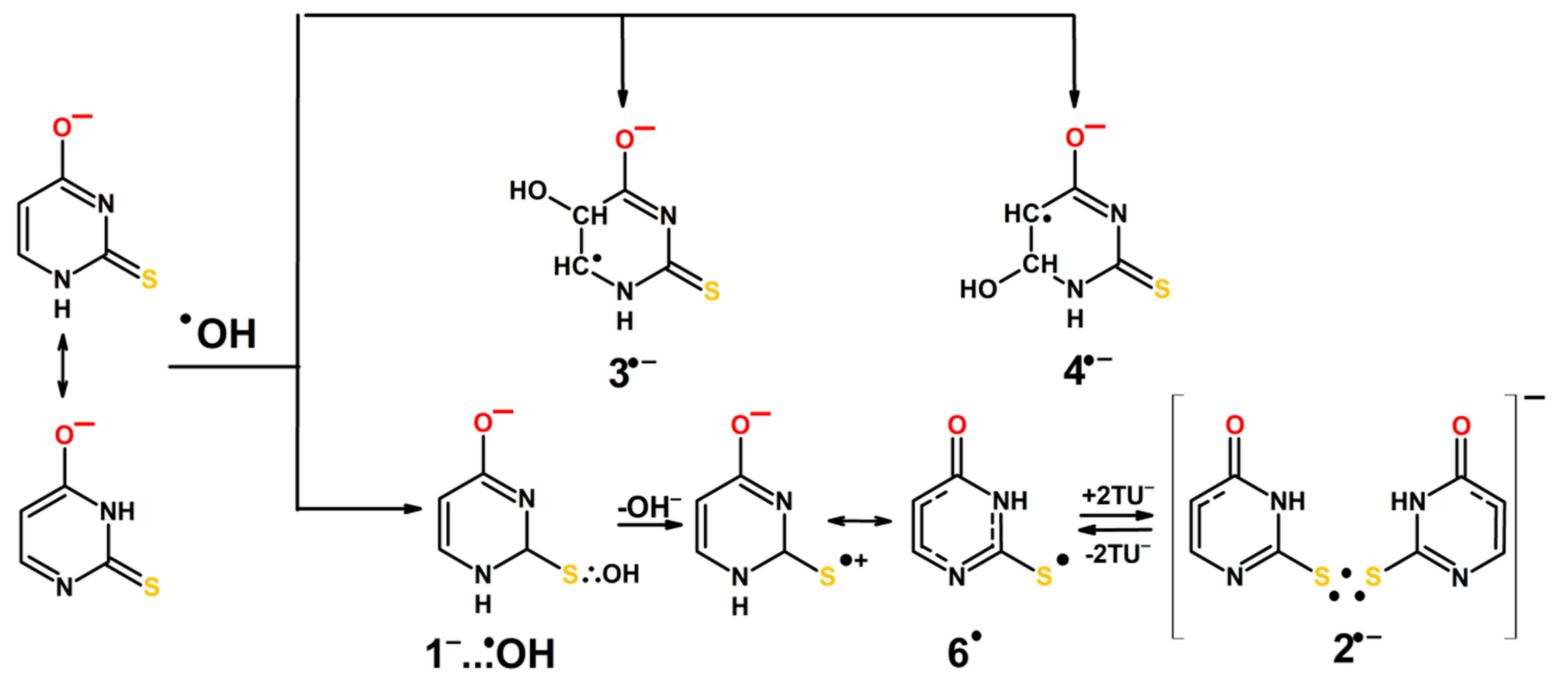
^●^OH-induced oxidation of 2-TU at pH above its pK_a._

On the basis of the identification of transients and complementary theoretical calculations, the mechanisms presented in Scheme 4 to Scheme 5 are proposed for the ^●^OH and ^●^N_3_-induced oxidation of 2-TU in aqueous solutions.

The initial steps are the additions of ^●^OH radical to C5=C6 double bond and to S8 atom yielding 3^●^ and 4^●^ radicals and hemibonded adduct to sulfur (1…^●^OH), respectively. Based on our calculations we can assume regioselectivity in addition to position C5 over C6 and in a consequence more efficient formation of radical 3^●^. Furthermore formation of 2c-3e OH adducts to sulfur should dominate based on computed energy profiles for ^●^OH addition reactions to neutral 2-TU.

The hemibonded adduct to sulfur (1…^●^OH) decays by separated coupled electron proton transfer leading to formation of 6^●^ or 7^●^ radicals. Since formation of radical 6^●^ is thermodynamically preferred (*vide* Table S2 in Supplementary Materials) this form is presented in the scheme. At higher 2-TU concentration 6^●^ radicals are converted into dimeric radical 2^●^, however, both radicals exist in an equilibrium (Scheme 4). The dimeric radical exists in neutral form since both substrates (6^●^ and 2-TU) are present in neutral forms, too.

At higher pH, the initial steps of ^●^OH reactions with 2-TU are practically the same and lead to the following species—3^●^^−^, 4^●^^−^ and 1^−^…^●^OH. The hemibonded adduct to sulfur is then converted to 6^●^. Similarly, as for pH = 4, at higher concentration of 2-TU radical 6^●^ is in an equilibrium with the dimeric radical anion (2^●^^−^). The anionic character of dimeric radical is due to the fact that at pH = 10, 2-TU exists in anionic forms (Scheme 5). Formation of 3^●−^ is even more preferable than formation of 4^●-^, in comparison to 3^●^ and 4^●^. In this case both lower activation barrier and lower ∆G favor its formation (Figure S10B,C; Table S2 in Supplementary Materials).

**Scheme 6:**
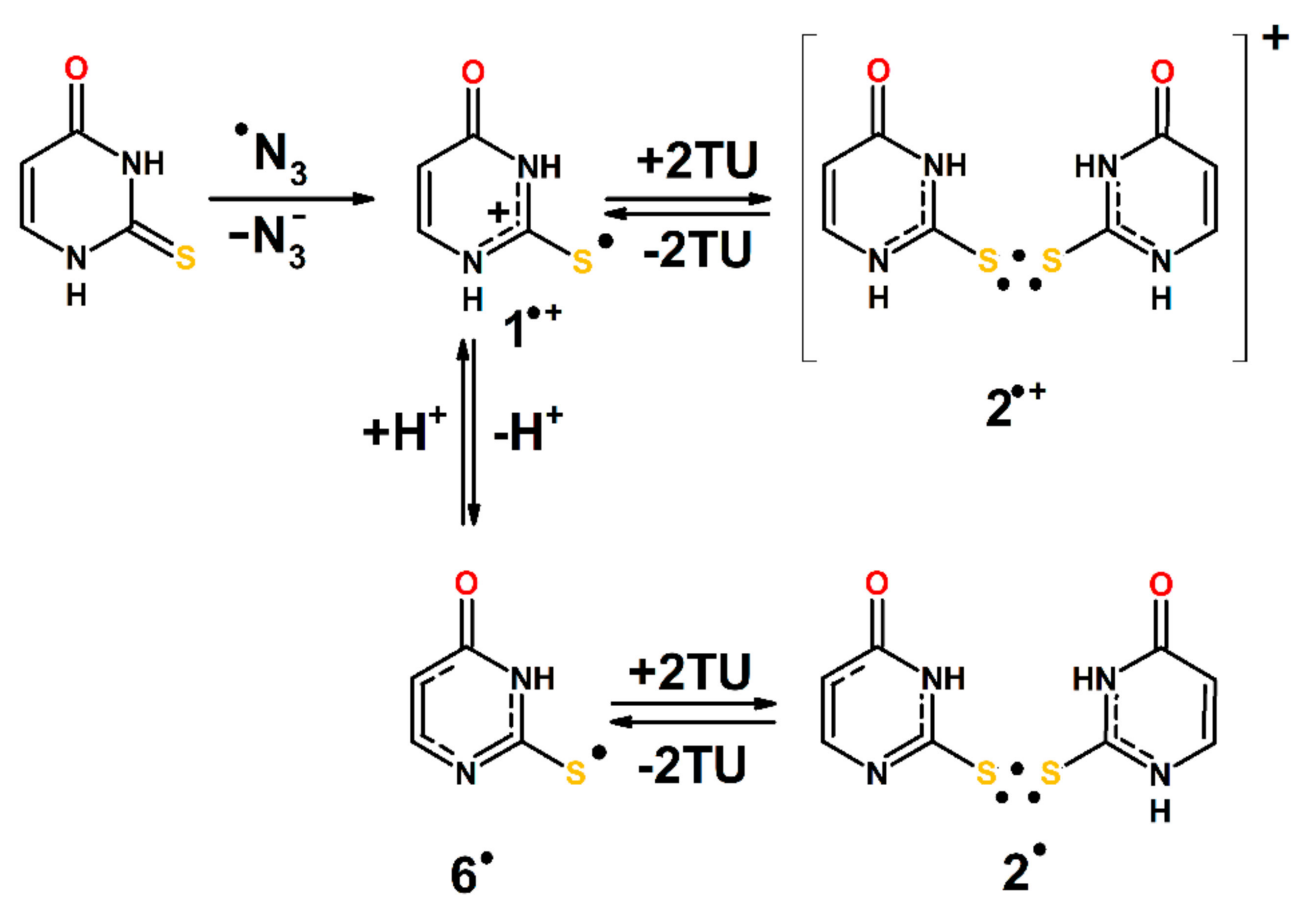
^●^N_3_-induced oxidation of 2-TU at pH below its pK_a._

Since azide radicals (^●^N_3_) are commonly considered as one-electron oxidants the initial step leads to the radical cation (1^●+^). Formation of this radical cannot be observed directly because its absorption spectrum is located in inconvenient observation area < 300 nm. In principle, the radical cation (1^●+^) could be a precursor of dimeric radical cation (2^●+^). Its spectral features are very similar to those showing by 2^●^ and 2^●^^−^ (Figure 8). However, one can assume another scenario. Radical cation (1^●+^) can also undergo fast deprotonation to radical 6^●^ which further undergo transformation to dimeric radical 2^●^. One important observation strongly supports this reaction pathway. At low concentration of 2-TU, a transient absorption spectrum with λ_max_ = 320 nm is observed which can be tentatively assigned to radical 6^●^ (Figure 7A) Moreover, the transient absorption in the vicinity of λ = 350 nm observed at high concentration of 2-TU (Figure 7B) can be also tentatively assigned to radical 6^●^. Since the pseudo-first order rate constant of 6^●^ radical decay matches the pseudo-first order rate constant growth measured at the λ_max_ assigned to the absorption bands of dimeric radicals, this is a strong support that radical 6^●^ is a direct precursor of 2^●^ (Scheme 6).

## 4. Materials and Methods

### 4.1. Chemicals

2-Thiouracil (2-TU) (≥99% purity) and sodium azide (NaN_3_) (≥99.5% purity) were purchased from Sigma-Aldrich (St. Louis, MO, USA) and used without further purification. Nitrous oxide (N_2_O) > 98% was from Messer (Warsaw, Poland).

### 4.2. Preparation of Solutions

All solutions were made with water triply distilled provided by a Millipore Direct-Q 3-UV system. The pH was adjusted by the addition of NaOH (≥99% purity) or HClO_4_ (70%, 99% purity), both form Sigma-Aldrich (St. Louis, MO, USA). Prior to irradiation, the samples were purged gently with N_2_O for 30 min. per 200 mL volume.

### 4.3. Pulse Radiolysis

Pulse radiolysis experiments with time-resolved UV-vis optical absorption detection were carried out at the Institute of Nuclear Chemistry and Technology (INCT) in Warsaw, Poland and the Notre Dame Radiation Laboratory (NDRL), Notre Dame, IN, USA. In the INCT, the linear electron accelerator (INCT LAE 10) delivering 10 ns pulses with electron energy about 10 MeV was applied as a source of irradiation. The 150 W xenon arc lamp E7536 (Hamamatsu Photonics K.K) was used as a monitoring light source. The respective wavelengths were selected by MSH 301 (Lot Oriel Gruppe) motorized monochromator/spectrograph with two optical output ports. The time dependent intensity of the analyzing light was measured by means of photomultiplier (PMT) R955 (Hamamatsu). A signal from detector was digitized using a WaveSurfer 104MXs-B (1 GHz, 10 GS/s, LeCroy) oscilloscope. A detailed description of the experimental setup has been given elsewhere along with the basic details of the equipment and the data collection system [93,94]. Absorbed doses per pulse were on the order of 10–20 Gy (1 Gy = 1 J·kg^–1^). Experiments were performed with a continuous flow of sample solutions at room temperature (~22 °C). In order to avoid photodecomposition and/or photobleaching effects in the samples, the UV or VIS cut-off filters were used. However, no evidence of such effects was found within the time domains monitored. Water filter was used to eliminate near IR wavelengths. Absorption intensities are presented in *G* × ε, where *G* is radiation-chemical yield (µM J^−1^) of given species and ε represents molar absorption coefficient. This value is directly proportional to the absorbance.

At the NDRL, pulse radiolysis experiments were carried out using the 8-MeV linear accelerator (LINAC). Following 8 ns, ~15 Gy electron pulse, transient optical absorption signals were recorded in UV−visible range using two different detection systems. First, single channel PMT/monochromator detection system was described elsewhere [95]. Second, multichannel system with nanosecond response recorded array of 24 monochromatic kinetic signals on all input channels of 6 synchronously triggered Tektronix oscilloscopes. The oscilloscopes were connected to an array of equivalent 24 silicon photodiode/amplifier detectors. Each of the detectors was optically coupled with different monochromatic line generated at the exit focal plane of SpectraPro 2150 spectrograph (Princeton Instruments) using a custom bundle of 24 fused silica fiber optics. The probe light source for both systems was a 1000 W xenon lamp pulsed to high current for 2 milliseconds. Variation of the solute concentration was accomplished using two HPLC pumps, one of which was pumping N_2_O saturated 2mM stock solution of 2-TU and the other was filled with N_2_O saturated water at the same pH as the 2-TU solution. The solutions from the pumps were mixed at a tee connection before the inlet of the flow cell. By varying the flow rates of the two pumps, with constant total flow of 3 mL/min, the concentration of 2-TU could be easily remotely changed/controlled.

At both pulse radiolysis set-ups, the dosimetry was based on N_2_O-saturated solutions containing 10 mM KSCN, taking a radiation chemical yield of G = 0.635 μmol J^–1^ and a molar absorption coefficient of 7580 M^–1^·cm^–1^ at λ = 472 nm for the (SCN)_2_^●^^−^ radical [96].

For time-resolved conductivity measurements, the conductivity apparatus was used which allows high-precision conductometric measurements over a pH range from 3 to 6. In the current experiments, pH was restricted to 4.1. A detailed description of the conductivity apparatus along with the measuring cell has been given elsewhere [97]. The dosimetry was achieved using acidic (pH = 4.1), aqueous solution saturated with methyl chloride (CH_3_Cl). In this dosimeter system, pulse irradiation yields H^+^ and Cl^−^ with *G*(H^+^) = *G*(Cl^−^) = 0.285 μmol J^–1^. The respective equivalent conductivities at 18 °C were taken as Λ(H^+^) = 315 S cm^2^ equiv^−1^ and Λ(Cl^−^) = 65 S cm^2^ equiv^−1^ [98].

### 4.4. Theoretical Procedures

The theoretical calculations were performed using the Gaussian 09 and Gaussian 16 program package [99]. One computational method have been used in this work for the geometry optimizations, ground state reactivities and excited state calculations based on the range separated hybrid (RSH) functionals ωB97x [100] and correlation consistent basis sets of triple ζ type, augmented with diffuse functions, denoted aug-cc-pVTZ [101,102]. The hydration effects were taken into consideration using a polarized continuum model (IEFPCM) [103]. The local minima were verified by frequency calculations. The Mulliken scheme was used to obtain spin density distribution. The transition states (TS) were located by the Synchronous Transit-Guided Quasi-Newton approach (QST3 method). The genuineness of the transition states in each case was ensured by the presence of one imaginary frequency related to either the stretching of the C-O bond (for OH addition) or H-O bond (for H abstraction) that connects the ^•^OH and 2-TU neutral or anionic reactants. Intrinsic reaction coordinate (IRC) calculations have been carried out from the TS, leading to OH adducts (at C5 and C6 positions of neutral 2-TU) and the pre-reactive complex (RC^•^). IRC calculations were also carried for H abstraction reactions from N3 and N1 positions in neutral and anionic forms of 2-TU. In case of anionic 2-TU no effort was taken to find optimized structures of pre-reactive complexes for OH addition as preliminary potential energy surface scans showed lack of such a complex and its eventual existence did not seem relevant to most of important findings of the work presented. Electronic transition energies and oscillator strengths were calculated by the time-dependent DFT (TD-DFT) method. DFT ωB97x method was proven to be very satisfactory in characterization of ground state geometries, harmonic vibrational frequencies, dissociation energies and absorption maxima of 2c-3e intermediates of (SCN)_2_^●−^ and (SeCN)_2_^●−^ [78,79]. Similar good performance of ω B97x based TD-DFT method was documented earlier [104,105]. It compared well with higher levels of theory in modeling ground-state reactivity and activation barriers for ^●^OH addition to double bonds in vacuum [106]. However, it could differ from them in obtaining proper activation barriers in certain cases in PCM solvent and/or explicit water molecules [107]. Since ^●^OH addition leading to formation of 2c-3e SO bonds is essentially barrierless [90] application of this relatively economic computational method serves as a good compromise in quantitative estimation of relative pathways in molecular systems where H abstraction, OH addition to double bonds and SO hemibond formation can occur simultaneously. We performed verification of accuracy of ωB97x functional in determining vertical excitation energies of OH adducts to C5 and C6 positions in 2-TU. In addition to that we compared CASPT2 level transitions obtained previously for ^●^OH adducts to uracil with transitions calculated using method applied in our studies of 2-TU. Using the starting geometries of OH adducts to uracil taken from the Supporting Information in Reference [108] we re-optimized them at the level of ωB97x/aug-cc-pvtz (PCM) and computed their absorption maxima. For most stable ground state structures—adduct U5OH (Hext)—we obtained a second transition at 4.13 eV versus CASPT 4.39 eV (−0.26 eV or +18 nm) and for adduct U6OH (Hint) we obtained transition at 3.56 eV versus CASPT 3.05 eV (+0.51eV or −58 nm). This may seem like a big difference but one needs to point out that both calculations should be referred to the experimental value at 3.26 eV. U6OH adduct CASPT computed transition was shifted from the experimental value by +27 nm and TD-DFT wB97x/aug-cc-pvtz computed transition was shifted −31 nm from the experimental value of 380 nm. For a broad spectrum with half width of ~1 eV we would consider both values as quite satisfactory. Our TD-DFT calculations predicted transition for U5OH to occur at ~299.9 nm versus 282 nm obtained by CASPT. These values are qualitatively quite comparable to previous findings obtained at much higher computational cost [108]. It has been documented that DFT and TD-DFT results always display some spin contamination, namely, the mean value of the S^2^ spin operator for the ground and excited states differs from the theoretical result < S^2^ > = S(S + 1) = 0.75. This is due to the single determinant form of the wave function in DFT [109]. Consequently, these states may no more be called spin doublet states but spin-contaminated states where only the quantum number Ms = ±1/2 is ensured. These contaminated states ψ_C_ may be considered a combination of the doublet state ψ_D_ and quartet state ψ_Q,_ ψ_C_ = ψ_D_ + εψ_Q_ (with Ms = ±1/2). Analyzing results of our TD-DFT computations we noticed that most relevant transitions (with the highest oscillator strengths) in all considered 2c-3e intermediates never had <S^2^> value higher than 0.77, hence we considered them not spin contaminated. On the other hand, thiyl radicals as well as OH adducts to C5 and C6, which were found to have much lower oscillator strengths in our experimental spectral range of interest have shown <S^2^> values extending up to 1.0 for the strongest transitions and >1 for the transitions with oscillator strengths 1–2 orders of magnitude lower than these strongest transitions. Therefore, results of computations of excited states in these intermediates are affected by spin contamination and in order to get more accurate predictions of their absorption spectra their ground and excited states should be treated using higher level computational methods which was beyond interest in our current studies. This was justified by the fact that contribution of these intermediates to the observed spectra was relatively minor. We still decided to include these spectra to all computed 2c3e intermediates for the qualitative comparison. All computed UV-Vis spectra were generated using the default setting of GaussView 6 in which peaks assume a Gaussian band shape with half widths of 0.4eV [110].

## 5. Conclusions

In the current paper, we provided an experimental proof supported by the TD-DFT calculations that character of primary and secondary reactive intermediates depends on the concentration of 2-thiouracil and character of the oxidant (^●^OH vs. ^●^N_3_). At low concentration of 2-TU, ^●^OH-adducts to the double bond at C5 position (3^●^) and 2c-3e bonded ^●^OH adducts to sulfur (1…^●^OH) are dominant species. Their relative contribution seems to depend on the individual rate constants of ^●^OH addition to the double bonds and sulfur atom, respectively. In turn, at high concentration of 2-TU, beyond contribution of 3^●^ radicals, contribution of dimeric 2c-3e S-S-bonded radicals in neutral form (2^●^) has to be taken into account. Their direct precursors are thiyl-type radicals (6^●^) which are formed from 1…^●^OH radicals via separated coupled electron proton transfer. Dimeric 2c-3e S-S-bonded radicals are also formed at pH above the pK_a_ of 2-TU but they have anionic character (2^●−^) owing to the anionic form of 2-TU. In turn, ^●^N_3_-induced oxidation of 2-TU occurs via radical cations with maximum spin location on the sulfur atom (1^●+^) which subsequently undergo deprotonation at N1 atom leading to thiyl-type radicals (6^●^), direct precursors of dimeric radicals (2^●^).

## Supplementary Materials

The following are available online at https://www.mdpi.com/1420-3049/24/23/4402/s1, Figure S1: Transient absorption spectra recorded in N_2_O-saturated unbuffered aqueous solution at pH = 4 containing 0.2 and 0.5 mM of 2-TU, Figure S2: Absorption spectra recorded in aqueous solutions containing 2-TU at pH = 4 and pH = 10, Figure S3: Time profiles representing growth and decay of transient absorptions at λ = 420 nm at pH = 4 and at pH = 10 at various concentration of 2-TU, Figure S4: Solution phase (PCM) optimized geometries of monomeric type transients formed at pH lower than pK_a_ of 2-TU, Figure S5: Solution phase (PCM) optimized geometries of monomeric type transients formed at pH higher than pK_a_ of 2-TU, Figure S6: Solution phase (PCM) optimized geometries of 2c-3e SS dimers. Figure S7: Computed SCF spin density isosurfaces of various 2c-3e intermediates. Figure S8: Relative energy profile in aqueous phase (PCM) for the H abstraction from neutral 2-TU via 2c-3e OH adducts. Figure S9: Relative energy profiles in aqueous phase (PCM) for the H abstraction and ^•^OH addition from/to 2-TU. Figure S10: Relative energy profiles in aqueous phase (PCM) for the ^•^OH addition (and H abstraction) reactions to/from 2-TU monoanions. Figure S11: TD-DFT calculated absorption spectra of potential transients produced in ^●^OH-induced oxidation of 2-thiouracil (2-TU) in water at pH higher than its pK_a_. Table S1: Thermochemistry values for the reactants and products optimized structures. Table S2: Free energies of reactions^*^ of **^•^**OH addition to 2-TU at pH 4 and 10. Table S3: Cartesian coordinates of the studied structures.
