# Supplementary material for: Radiation Induced One-Electron Oxidation of 2-Thiouracil in Aqueous Solutions
Source: Molecules. 2019 Dec 2;24(23):4402. doi: 10.3390/molecules24234402 (PMC6930642; doi:10.3390/molecules24234402)
Supplement: Supplementary file 1 [file molecules-24-04402-s001.pdf]

Supplementary Materials

Radiation induced one-electron oxidation of 2-thiouracil in aqueous solutions

Konrad Skotnicki *, Katarzyna Taras-Goslinska, Ireneusz Janik *, and Krzysztof Bobrowski

* Correspondence: k.skotnicki@ichtj.waw.pl; Tel: +48-22-5041292; ijanik@nd.edu

Table of contents:

Figure S1	page 2
Figure S2	page 2
Figure S3	page 3
Figure S4	page 4
Figure S5	page 5
Figure S6	page 6
Figure S7	page 7–8
Figure S8	page 9
Figure S9	page 10
Figure S10	page 11
Figure S11	page 12
Table S1	page 13
Table S2	page 14
Table S3	page 15–25

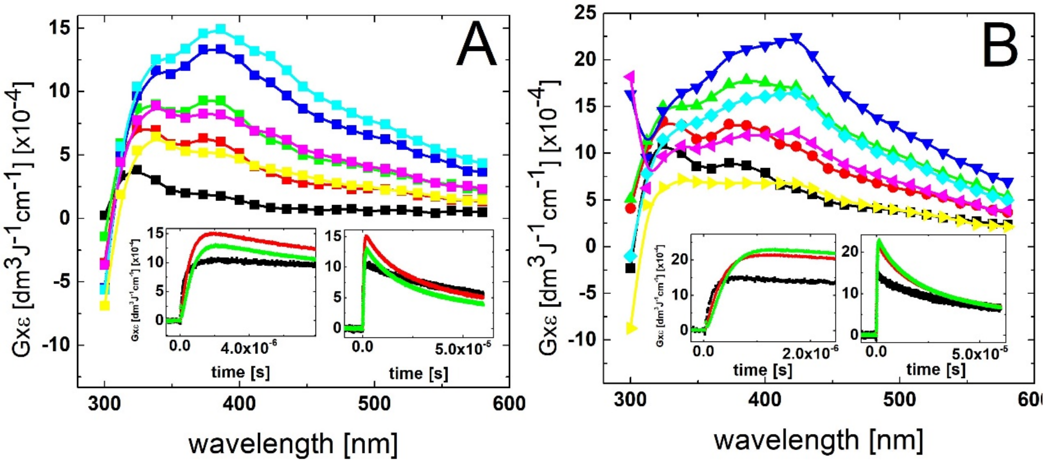

Figure S1. Transient absorption spectra recorded in N_2O-saturated unbuffered aqueous solution at pH = 4 containing (**A**) 0.2 mM of 2-TU 100 ns (■), 300 ns (■), 500 ns (■), 1 μs (■), 2 μs (■), 25 μs (■) and 60 μs (■) after electron pulse. <u>Inserts</u>: time profiles representing growth (left) and decay (right) of transient absorptions at λ = 338 nm (■), 386 nm (■) and 426 nm (■); (**B**) 0.5 mM of 2-TU 200 ns (■), 300 ns (■), 500 ns (■), 1 μs (■), 10 μs (■), 25 μs (■) and 60 μs (■) after electron pulse. <u>Inserts</u>: time profiles representing growth (left) and decay (right) of transient absorptions at λ = 338 nm (■), 386 nm (■) and 426 nm (■).

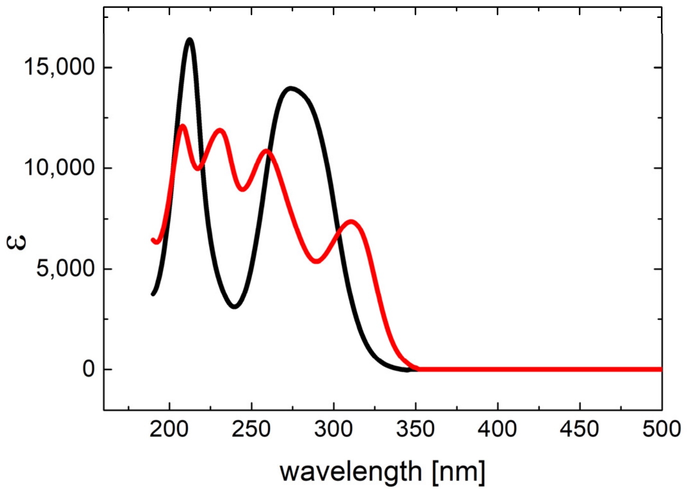

Figure S2. Absorption spectra recorded in aqueous solutions containing 2-thiouracil at pH = 4 (black line) and pH = 10 (red line).

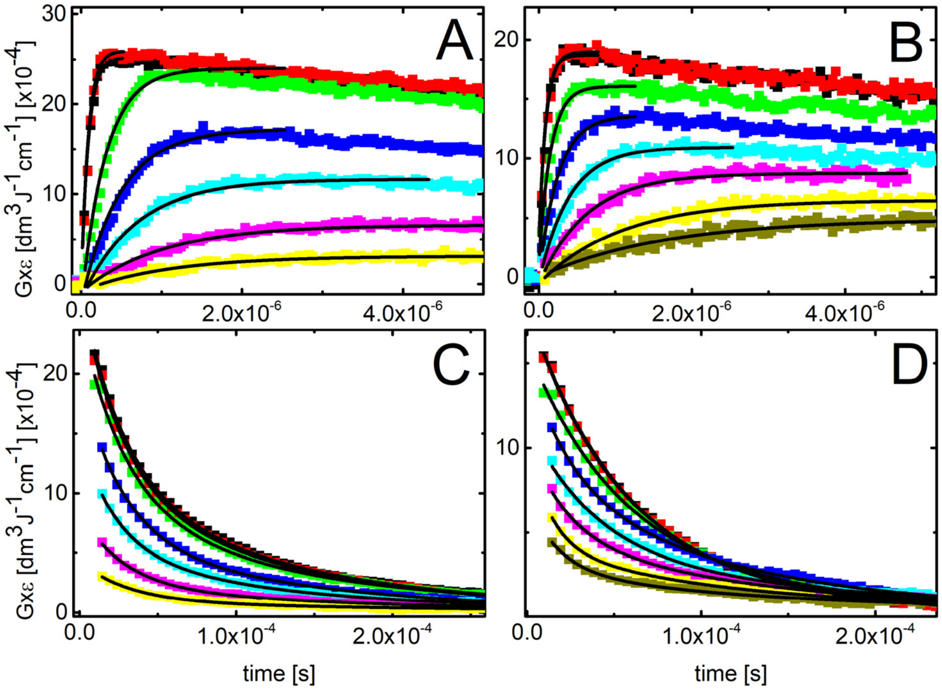

Figure S3. Time profiles representing growth (top panels) and decay (bottom panels) of transient absorptions at λ = 420 nm at pH = 4 (**A** and **C** panels) and at pH = 10 (**B** and **D** panels) at various concentration of 2-TU: A and C panels: 0, 047 mM (■), 0.094 mM (■), 0.188 mM (■), 0.375 mM (■), 0.75 mM (■), and 3 mM (■, ■); B and D panels: 0.047 mM (■), 0.094 mM (■), 0.188 mM (■), 0.375 mM (■), 0.75 mM (■), 1.5 mM (■), and 3 mM (■, ■). .

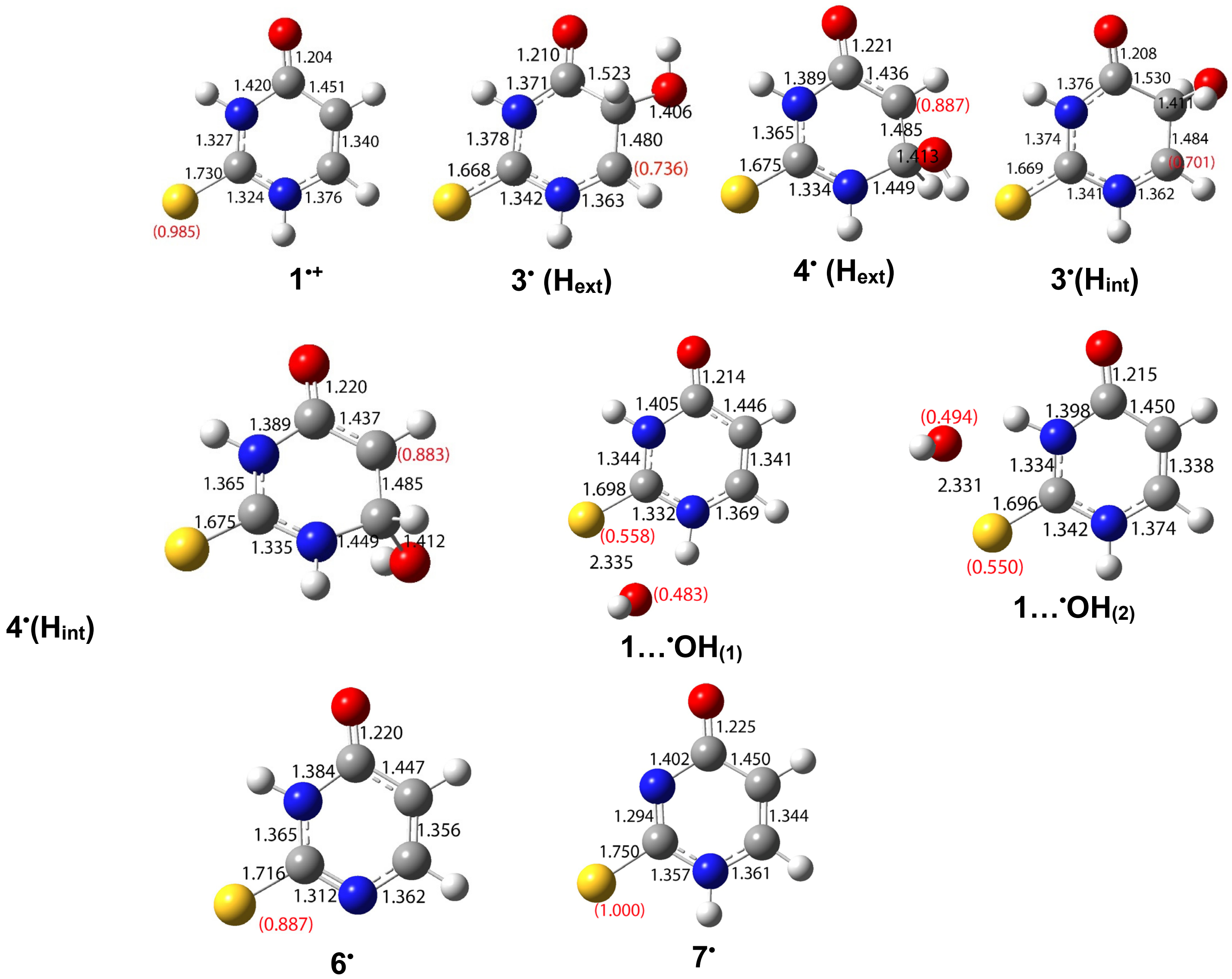

Figure S4. Solution phase (PCM) optimized geometries (ωB97x/aug-cc-pvtz) of monomer type transients expected to be formed in solutions at pH = 4 (lower than the first pK_a of 2-thiouracil). Selected bond lengths are in Å units. Maximum spin population is given in red color in parentheses.

.

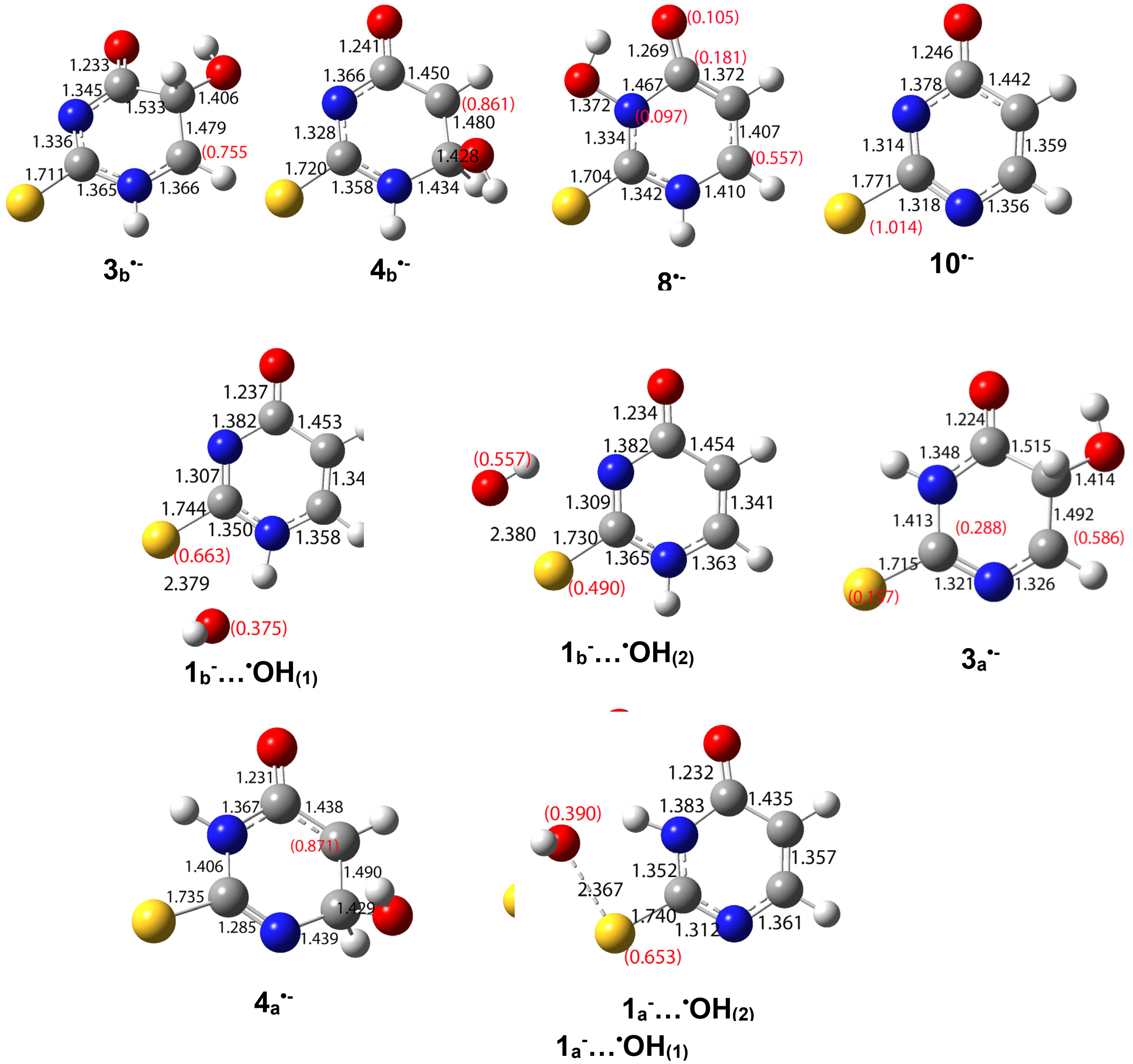

Figure S5. Solution phase (PCM) optimized geometries (ωB97x/aug-cc-pvtz) of monomer type transients expected to be formed in solutions at pH = 10 (higher than the first pK_a of 2-thiouracil). Selected bond lengths are in Å units. Maximum spin population is given in red color in parentheses.

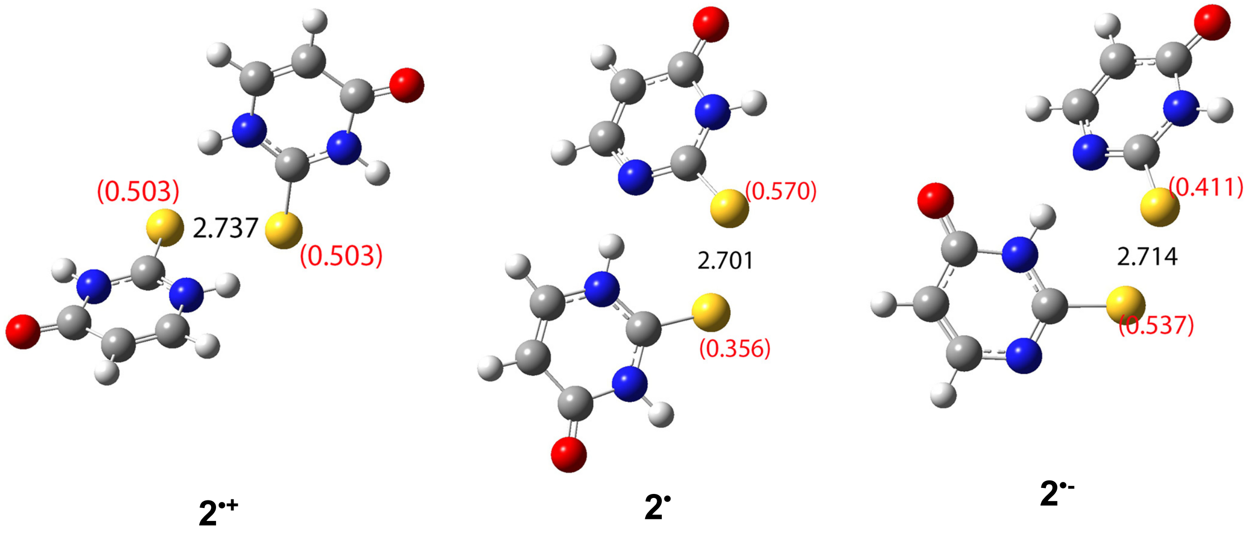

Figure S6. Solution phase (PCM) optimized geometries (ωB97x/aug-cc-pvtz) of 2c-3e SS dimers; SS bond lengths are in Å units. Maximum spin population is given in red color in parentheses.

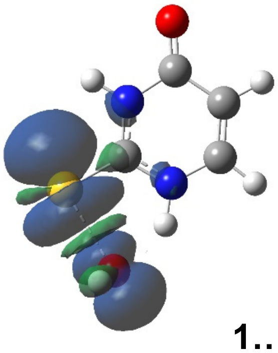

$\mathbf{1}\ldots{}^{\bullet}\mathrm{OH}_{(1)}$

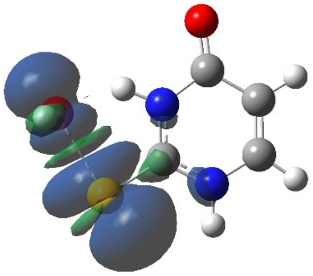

$\mathbf{1}\ldots{}^{\bullet}\mathrm{OH}_{(2)}$

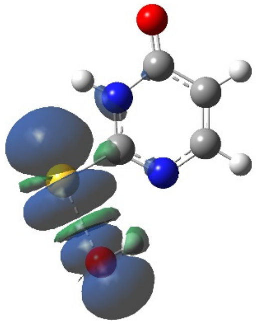

$\mathbf{1}_{a}^{-}\ldots{}^{\bullet}\mathrm{OH}_{(1)}$

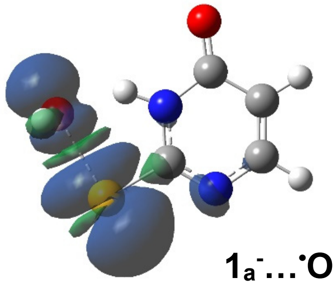

$\mathbf{1}_{a}^{-}\ldots{}^{\bullet}\mathrm{OH}_{(2)}$

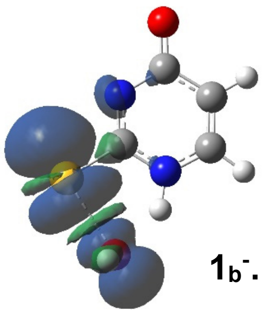

$\mathbf{1}_{b}^{-}\ldots{}^{\bullet}\mathrm{OH}_{(1)}$

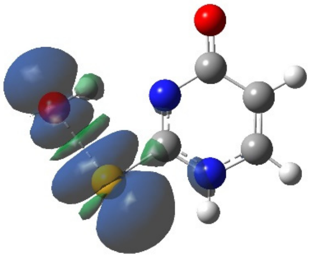

$\mathbf{1}_{b}^{-}\ldots{}^{\bullet}\mathrm{OH}_{(2)}$

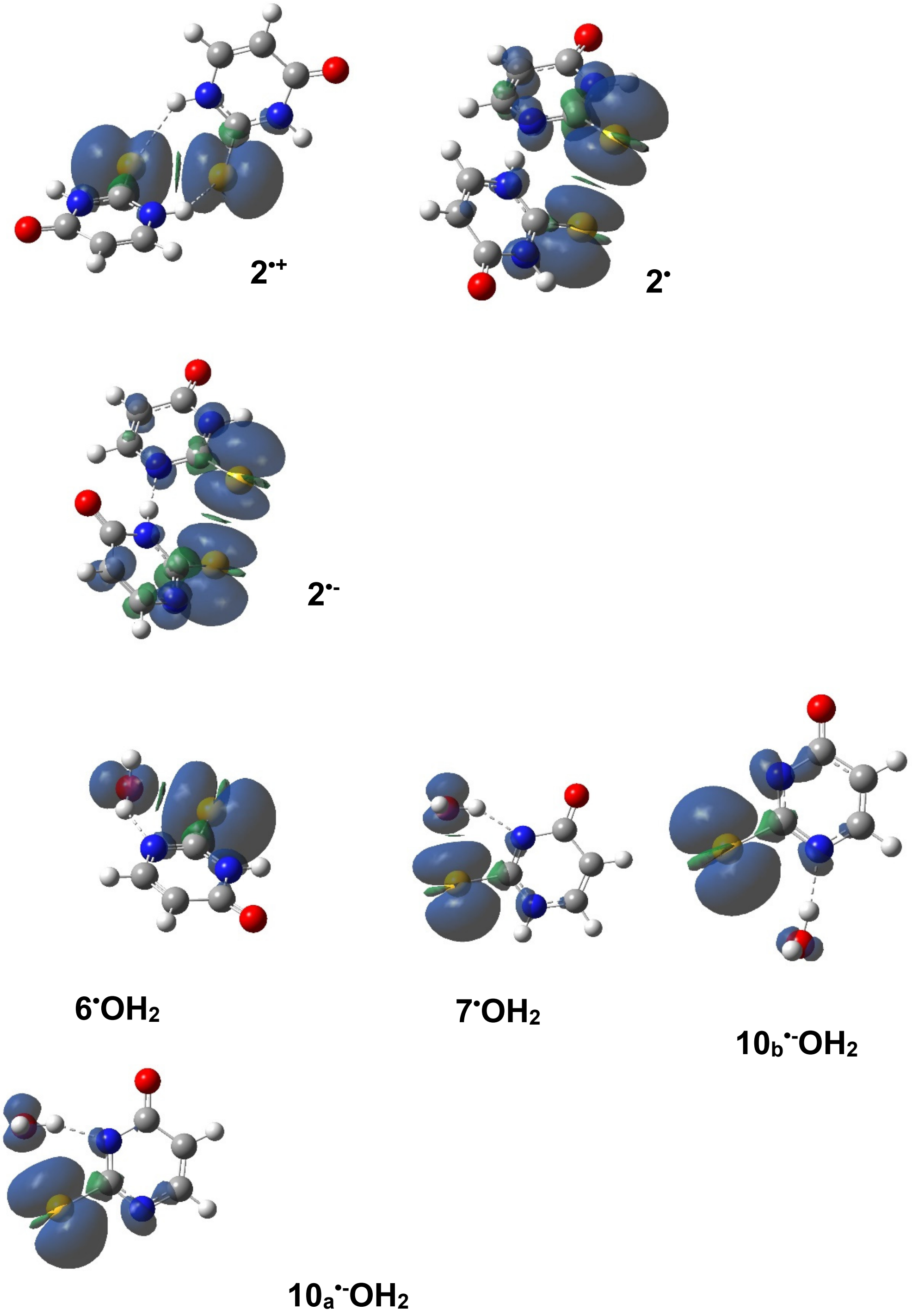

Figure S7. Computed SCF spin density = 0.001 bohr^{-3} isosurfaces of various 2c-3e intermediates produced in •OH induced oxidation of 2-TU at pH = 4 and 10.

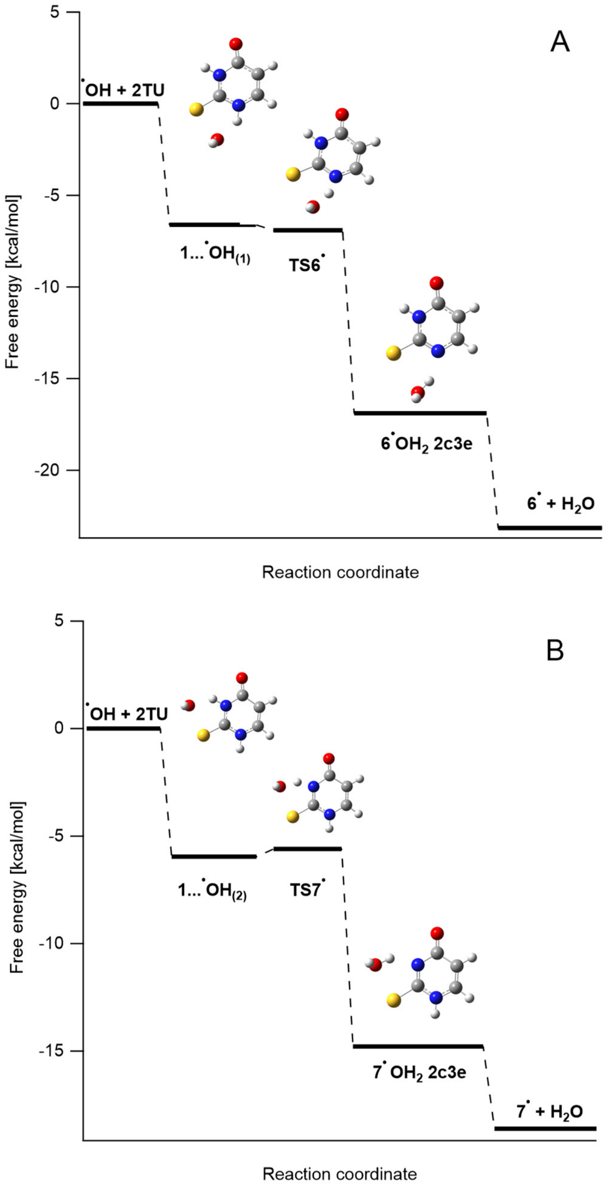

Figure S8. Relative energy profiles in aqueous phase (PCM) for the H abstraction from neutral 2-TU at N1 (**A**) and N3(**B**) positions via 2c-3e OH adducts at ωB97x/aug-cc-pvtz level of theory.

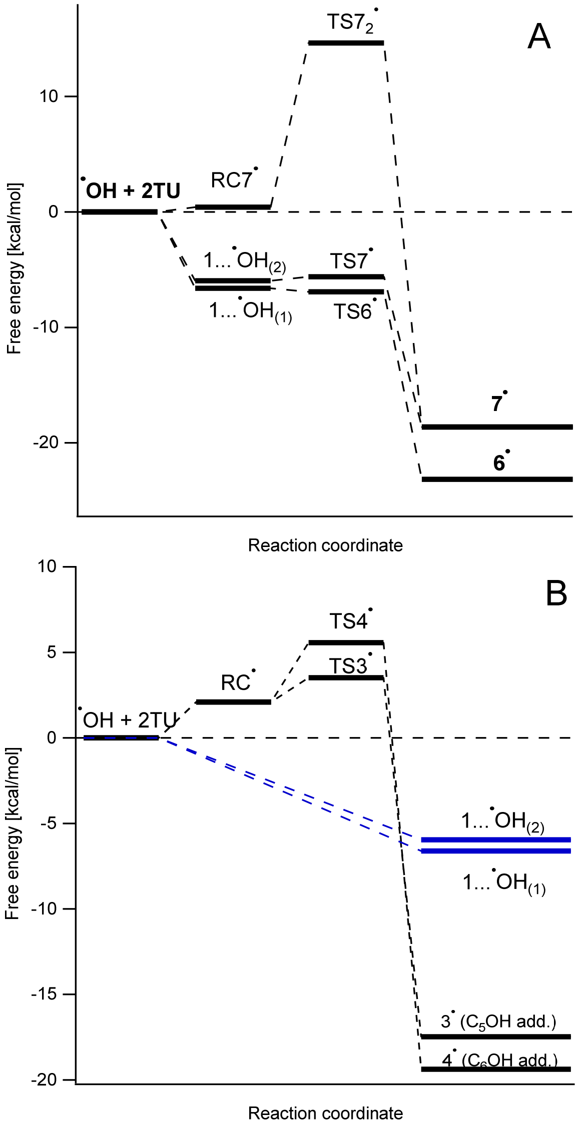

Figure S9. Relative energy profiles in aqueous phase (PCM) for H abstraction (A) and •OH addition (**B**) reactions from/to neutral 2-TU at ωB97x/aug-cc-pvtz level of theory.

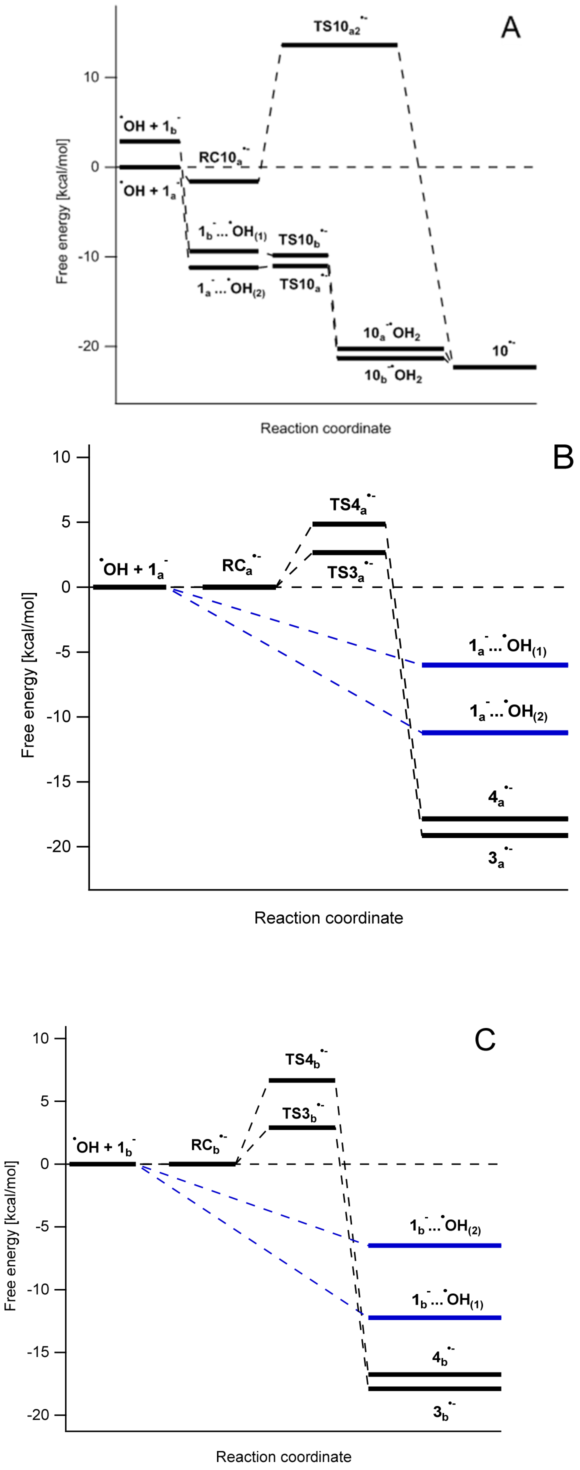

Figure S10. Relative energy profiles in aqueous phase (PCM) for H abstraction (A) and the •OH addition reactions (B and C) to/from 2-TU$^-$ monoanions (subscripts a or b indicate the site of deprotonation of neutral 2-TU as N1 or N3, respectively) at ωB97x/aug-cc-pvtz level of theory.

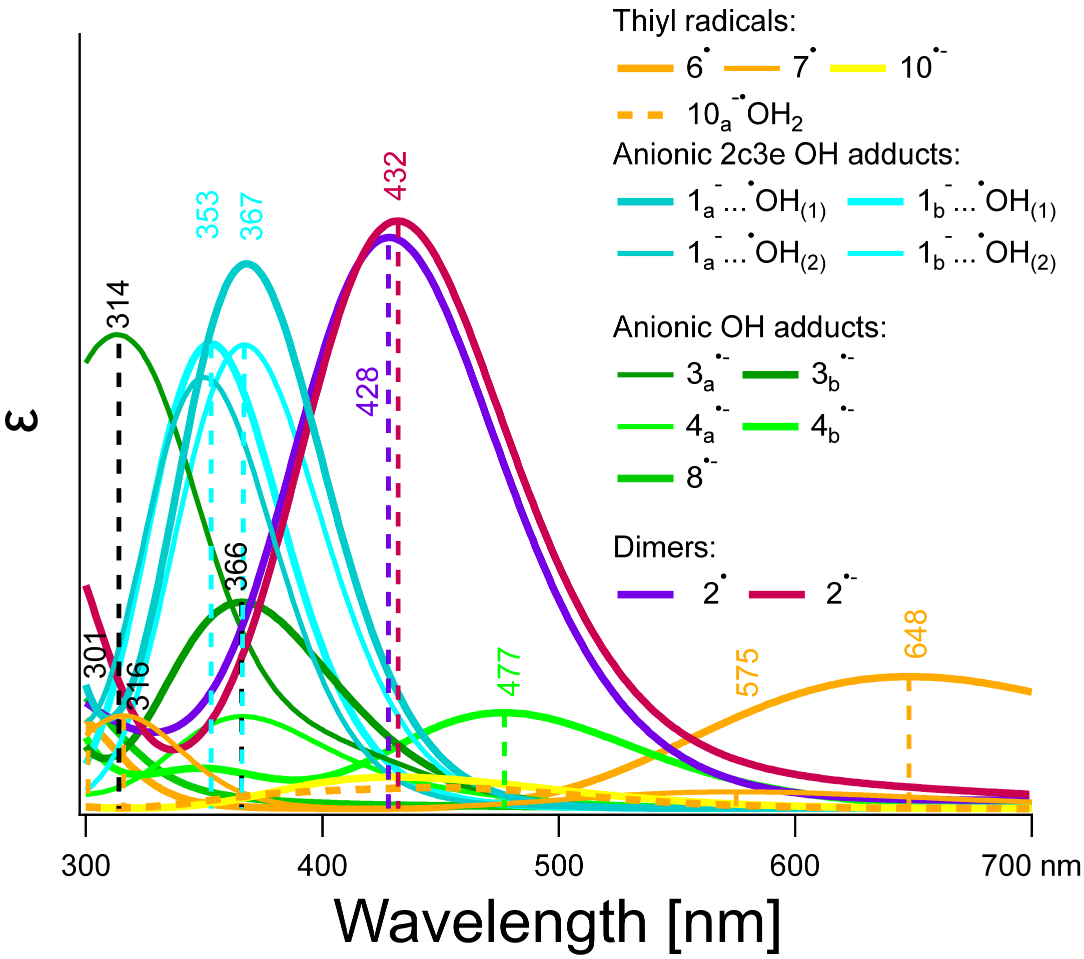

Figure S11. TD-DFT calculated absorption spectra of potential transients (see legend for symbols and Figures S4 and S5 for geometries) produced in •OH–induced oxidation of 2-thiouracil (2-TU) in water at pH = 10 (higher than its first pKa = 7.75).

Table S1. Thermochemistry values for the reactants, products, prereactive complexes, and transition states optimized structures (tight convergence with ultrafine grid) at the ωB97x/aug-cc-pvtz level of theory with water solvation modeled by IEFPCM. ε_0 - electronic energy, ε_{ZPE} - zero-point energy correction, E_{tot} - thermal correction to energy, H_{corr} – themal correction to enthalpy, G_{corr} - thermal correction to free energy.

Molecule	ε_0	ε_{ZPE}	E_{tot}	H_{corr}	G_{corr}	$\varepsilon_0 + \varepsilon_{ZPE}$	$\varepsilon_0 + E_{tot}$	$\varepsilon_0 + H_{corr}$	$\varepsilon_0 + G_{corr}$
					[Hartree]				
$^{\bullet}OH$	-75.744596	0.008589	0.010949	0.0119	-0.00833	-75.736007	-75.73365	-75.7327	-75.752927
H_2O	-76.448102	0.02147	0.024305	0.02525	0.00383	-76.426632	-76.423796	-76.422852	-76.444269
**1**	-737.8424036	0.086445	0.092753	0.093697	0.055325	-737.755958	-737.749651	-737.748707	-737.787078
1^{-}_{a}	-737.375525	0.072888	0.078988	0.079932	0.041939	-737.302637	-737.296537	-737.295593	-737.333586
1^{-}_{b}	-737.370885	0.07294	0.07912	0.080064	0.04185	-737.297945	-737.291765	-737.290821	-737.329035
$1^{\bullet+}$	-737.602067	0.085354	0.09143	0.092374	0.053749	-737.516713	-737.510637	-737.509693	-737.548319
$2^{\bullet+}$	-1475.476283	0.17454	0.188957	0.189902	0.12893	-1475.301743	-1475.28733	-1475.28638	-1475.347353
$2^{\bullet}$	-1475.0371	0.160706	0.174726	0.175671	0.11651	-1474.87639	-1474.8624	-1474.86143	-1474.920591
$2^{\bullet-}$	-1474.576029	0.147219	0.161258	0.162203	0.102184	-1474.42881	-1474.41477	-1474.41383	-1474.473845
$3^{\bullet}$ (H_{ext})	-813.633392	0.100136	0.108471	0.109415	0.065544	-813.533256	-813.524921	-813.523977	-813.567848
$3^{\bullet}$ (H_{int})	-813.63221	0.100552	0.108752	0.109696	0.06606	-813.531656	-813.52346	-813.522512	-813.56615
$4^{\bullet}$ (H_{ext})	-813.636492	0.100398	0.108639	0.109584	0.065835	-813.536093	-813.527852	-813.526908	-813.570657
$4^{\bullet}$ (H_{int})	-813.63739	0.100649	0.108713	0.109657	0.06652	-813.536743	-813.52868	-813.527735	-813.57087
$1…^{\bullet}OH_{(1)}$	-813.614772	0.098528	0.106944	0.107889	0.06424	-813.516243	-813.507827	-813.506883	-813.550531
$1…^{\bullet}OH_{(2)}$	-813.613767	0.09861	0.107086	0.108031	0.064273	-813.515156	-813.50668	-813.505736	-813.549494
$1_a^{-}…^{\bullet}OH_{(1)}$	-813.14618	0.085351	0.093841	0.094785	0.05012	-813.060823	-813.05233	-813.051389	-813.09606
$1_a^{-}…^{\bullet}OH_{(2)}$	-813.15511	0.084989	0.093315	0.094259	0.05074	-813.070125	-813.0618	-813.060854	-813.10437
$1_b^{-}…^{\bullet}OH_{(1)}$	-813.151981	0.084918	0.093285	0.094229	0.050529	-813.067063	-813.058696	-813.057752	-813.101452
$1_b^{-}…^{\bullet}OH_{(2)}$	-813.142041	0.085453	0.094006	0.09495	0.049729	-813.056588	-813.048035	-813.047091	-813.092312
$3_a^{\bullet-}$	-813.16927	0.086575	0.09457	0.095514	0.05229	-813.082696	-813.0747	-813.073757	-813.11698
$3_b^{\bullet-}$	-813.163469	0.086657	0.094506	0.09545	0.053	-813.076812	-813.068964	-813.068019	-813.110469
$4_a^{\bullet-}$	-813.16061	0.086611	0.09472	0.095664	0.05197	-813.074	-813.06589	-813.064948	-813.10865
$4_b^{\bullet-}$	-813.160776	0.086666	0.094799	0.095743	0.052095	-813.07411	-813.065977	-813.065032	-813.108681
$6^{\bullet}$	-737.1732	0.072322	0.078585	0.079529	0.040563	-737.100878	-737.094615	-737.093671	-737.132637
$7^{\bullet}$	-737.165978	0.071974	0.077841	0.078785	0.04059	-737.094004	-737.088137	-737.087193	-737.125387
$8^{\bullet-}$	-813.099416	0.084452	0.092111	0.093055	0.051217	-813.014964	-813.007306	-813.006362	-813.048199
$10^{\bullet-}$	-736.70548	0.05948	0.065641	0.066585	0.027672	-736.645999	-736.639838	-736.638894	-736.677807
$RC^{\bullet}$	-813.59673	0.096897	0.10647	0.107415	0.06005	-813.499828	-813.49026	-813.48931	-813.53667
$TS3^{\bullet}$	-813.59636	0.097169	0.105805	0.10675	0.06197	-813.499195	-813.49056	-813.489615	-813.5344
$TS4^{\bullet}$	-813.5944	0.09756	0.105799	0.106744	0.06326	-813.496841	-813.4886	-813.487657	-813.53114
$RC7^{\bullet}$	-813.60134	0.097785	0.106581	0.107525	0.062	-813.503555	-813.4948	-813.493814	-813.53934
$TS3a^{\bullet-}$	-813.13174	0.083868	0.091918	0.092863	0.04948	-813.047867	-813.03982	-813.038872	-813.08226
$TS4a^{\bullet-}$	-813.12891	0.08412	0.092098	0.093042	0.05015	-813.044791	-813.03681	-813.035869	-813.07876
$TS3b^{\bullet-}$	-813.1266	0.083889	0.09212	0.0931	0.0492	-813.0427	-813.034	-813.033489	-813.0773
$TS4b^{\bullet-}$	-813.12573	0.084081	0.092196	0.09314	0.04983	-813.04165	-813.03354	-813.032592	-813.07591
$TS6^{\bullet}$	-813.61188	0.094589	0.102506	0.10345	0.06086	-813.517286	-813.50937	-813.508426	-813.55102
$TS7^{\bullet}$	-813.6098	0.09465	0.102607	0.10355	0.06086	-813.515152	-813.5072	-813.506249	-813.548938
$TS7_2^{\bullet}$	-813.57713	0.094145	0.101977	0.102921	0.06042	-813.482985	-813.47515	-813.474209	-813.51671
$TS10a_2^{\bullet-}$	-813.11129	0.080194	0.087976	0.08892	0.04647	-813.031091	-813.02331	-813.022365	-813.06482
$TS10b^{\bullet-}$	-813.14897	0.08067	0.08864	0.089584	0.04676	-813.068297	-813.06033	-813.059383	-813.10221
$6^{\bullet}OH_2$	-813.628421	0.097692	0.107007	0.107951	0.06149	-813.530729	-813.52141	-813.52047	-813.566927

Table S2. Determination of free energies of reactions* of $^{\bullet}OH$ addition (or H abstraction) to (from) 2TU at pH = 4 and 10.

Reaction	$\Sigma(\varepsilon_0 + G_{corr})$ [Hartree]		$\Delta_r G^0$(298K) [kcal/mol]
	Reactants	Products	
$^{\bullet}OH + 1 \rightarrow 1 \ldots {}^{\bullet}OH_{(1)}$	-813.540005	-813.5505	-6.60
$^{\bullet}OH + 1 \rightarrow 1 \ldots {}^{\bullet}OH_{(2)}$	-813.540005	-813.5495	-5.95
$^{\bullet}OH + 1 \rightarrow 6^{\bullet} + H_2O$	-813.540005	-813.57691	-23.16
$^{\bullet}OH + 1 \rightarrow 6^{\bullet}OH_2$	-813.540005	-813.566927	-16.89
$^{\bullet}OH + 1 \rightarrow 7^{\bullet} + H_2O$	-813.540005	-813.56966	-18.61
$^{\bullet}OH + 1 \rightarrow 7^{\bullet}OH_2$	-813.540005	-813.563564	-14.78
$^{\bullet}OH + 1 \rightarrow TS6^{\bullet}$	-813.540005	-813.55102	-6.91
$^{\bullet}OH + 1 \rightarrow TS7^{\bullet}$	-813.540005	-813.548938	-5.61
$^{\bullet}OH + 1 \rightarrow TS7_2^{\bullet}$	-813.540005	-813.51671	14.62
$^{\bullet}OH + 1 \rightarrow RC7^{\bullet}$	-813.540005	-813.53934	0.42
$^{\bullet}OH + 1 \rightarrow 3^{\bullet}\ (H_{int})$	-813.540005	-813.566148	-16.40
$^{\bullet}OH + 1 \rightarrow 4^{\bullet}\ (H_{ext})$	-813.540005	-813.570657	-19.23
$^{\bullet}OH + 1 \rightarrow 4^{\bullet}\ (H_{int})$	-813.540005	-813.570871	-19.37
$^{\bullet}OH + 1 \rightarrow RC^{\bullet}$	-813.540005	-813.536672	2.09
$^{\bullet}OH + 1 \rightarrow TS3^{\bullet}$	-813.540005	-813.538077	3.52
$^{\bullet}OH + 1 \rightarrow TS4^{\bullet}$	-813.540005	-813.534398	5.56
$^{\bullet}OH + 1^{-}_{a} \rightarrow 1_a^{-} \ldots {}^{\bullet}OH_{(1)}$	-813.08651	-813.09606	-5.99
$^{\bullet}OH + 1^{-}_{a} \rightarrow 1_a^{-} \ldots {}^{\bullet}OH_{(2)}$	-813.08651	-813.10437	-11.20
$^{\bullet}OH + 1^{-}_{b} \rightarrow 1_b^{-} \ldots {}^{\bullet}OH_{(1)}$	-813.081962	-813.10145	-12.23
$^{\bullet}OH + 1^{-}_{b} \rightarrow 1_b^{-} \ldots {}^{\bullet}OH_{(2)}$	-813.081962	-813.09231	-6.49
$^{\bullet}OH + 1^{-}_{a} \rightarrow 10^{\bullet -} + H_2O$	-813.08651	-813.12208	-22.32
$^{\bullet}OH + 1^{-}_{b} \rightarrow 10^{\bullet -} + H_2O$	-813.081962	-813.12208	-25.17
$^{\bullet}OH + 1^{-}_{a} \rightarrow TS10_{a2}^{\bullet -}$	-813.08651	-813.06482	13.61
$^{\bullet}OH + 1^{-}_{b} \rightarrow TS10_{b}^{\bullet -}$	-813.081962	-813.10221	-12.7
$^{\bullet}OH + 1^{-}_{a} \rightarrow 3_a^{\bullet -}$	-813.08651	-813.117	-19.12
$^{\bullet}OH + 1^{-}_{a} \rightarrow 4_a^{\bullet -}$	-813.08651	-813.1086	-13.89
$^{\bullet}OH + 1^{-}_{b} \rightarrow 3_b^{\bullet -}$	-813.081962	-813.1105	-17.89
$^{\bullet}OH + 1^{-}_{b} \rightarrow 4_b^{\bullet -}$	-813.081962	-813.1087	-16.77
$^{\bullet}OH + 1^{-}_{a} \rightarrow TS3_a^{\bullet -}$	-813.08651	-813.08226	2.69
$^{\bullet}OH + 1^{-}_{a} \rightarrow TS4_a^{\bullet -}$	-813.08651	-813.0788	4.86
$^{\bullet}OH + 1^{-}_{b} \rightarrow TS3_b^{\bullet -}$	-813.081962	-813.07732	2.91
$^{\bullet}OH + 1^{-}_{b} \rightarrow TS4_b^{\bullet -}$	-813.081962	-813.0759	6.65
$1 + 1^{\bullet +} \rightarrow 2^{\bullet +}$	-1475.3354	-1475.3474	-7.50
$1 + 6^{\bullet} \rightarrow 2^{\bullet}$	-1474.9197	-1474.9206	-0.55
$1_a^{-} + 6^{\bullet} \rightarrow 2^{\bullet -}$	-1474.4662	-1474.4758	-6.040

CARTESIAN COORDINATES OF THE STUDIED STRUCTURES

Table S3. Cartesian x, y, and z coordinates (in Å) of the optimized structures (tight with ultrafine grid) at the ωB97x/aug-cc-pvtz level of theory with water solvation modeled by IEFPCM.

**1 (2TU)**			
C	0.84182900	1.71442500	-0.00000100
C	1.84393200	0.82615200	-0.00001800
H	0.99907800	2.78268500	-0.00000300
H	2.87643400	1.13463100	-0.00004200
C	1.53526600	-0.58714300	-0.00000200
O	2.33715000	-1.50148300	0.00002500
C	-0.85482300	0.02165100	-0.00000100
S	-2.45886400	-0.44068600	0.00000000
N	0.16473200	-0.87015000	-0.00002200
H	-0.08957600	-1.84725300	-0.00003600
N	-0.46978500	1.31789900	0.00001900
H	-1.20316500	2.00802600	0.00003800

**1^{-}_{a} (2TU$^{-}_{a}$, 2TU deprotonated at N_1)**			
C	0.81492800	1.71389600	-0.00000100
C	1.83212400	0.81225500	-0.00000100
H	1.04515700	2.77458100	-0.00000200
H	2.86864800	1.11358000	0.00000000
C	1.50875100	-0.58103600	-0.00000400
O	2.29566900	-1.53015800	0.00000000
C	-0.83982300	0.13969300	0.00000700
S	-2.46687200	-0.41220700	-0.00000200
N	0.14355500	-0.81619700	0.00000200
H	-0.14925400	-1.78138200	0.00000200
N	-0.50867400	1.41919000	0.00000000

**1^{-}_{b} (2TU$^{-}_{b}$, 2TU deprotonated at N_3)**			
C	-0.81887700	1.70252700	-0.00000600
C	-1.81944400	0.80957600	-0.00000300
H	-0.96432800	2.77343900	-0.00002000
H	-2.85291300	1.12031300	-0.00001500
C	-1.49174500	-0.60722700	0.00000100
O	-2.37561300	-1.47528400	-0.00000800
C	0.78914300	-0.06045900	0.00003300
S	2.46673700	-0.45120300	-0.00001100
N	-0.16267100	-0.96934300	0.00001000
N	0.47247200	1.27341400	0.00000700
H	1.23128600	1.93276400	-0.00000200

**$1^{\bullet+}$**			
C	-0.83479500	1.72469500	-0.00000200
C	-1.83385000	0.83172500	-0.00000200
H	-0.98365900	2.79296400	-0.00000500
H	-2.86467200	1.14822300	-0.00000700
C	-1.55305200	-0.59139300	0.00000500
O	-2.34775900	-1.49533400	-0.00000100
C	0.80845300	0.02664500	0.00000100
S	2.47403700	-0.44273900	-0.00000100
N	-0.16244500	-0.87752200	0.00000100
H	0.09775500	-1.85658000	-0.00000400
N	0.47734400	1.30888900	0.00000300
H	1.22323700	1.99228600	0.00000700

3•			
C	-0.66820500	-1.55769500	-0.00479500
C	-1.61365700	-0.47754900	-0.36706300
H	-0.97118800	-2.58966000	0.04580900
C	-1.02833900	0.89784000	-0.07629400
O	-1.73819000	1.86769700	0.06096300
C	1.22773700	-0.07024600	0.00309300
S	2.87682500	0.17865600	0.04067400
N	0.33940700	0.98233300	-0.02715100
N	0.66800200	-1.28983600	0.02957500
H	1.31527600	-2.05927900	0.10783700
H	0.73913100	1.90635900	0.04297700
H	-1.80216700	-0.48797500	-1.45387800
O	-2.82340100	-0.66028900	0.32674400
H	-3.37460900	0.11122800	0.15820700

4•			
C	-1.10973100	-1.25475700	0.38818500
C	-1.08715800	1.27276700	0.01991700
O	-1.62548600	2.35956700	-0.11921200
C	1.01039800	0.00368500	0.06082600
S	2.68029700	0.02787100	-0.06814100
N	0.28161400	1.13328200	-0.17403100
N	0.32999200	-1.08905500	0.41387600
H	0.88752000	-1.90717700	0.59723100
H	0.79806300	1.96372700	-0.41872700
H	-1.39518000	-1.81179300	1.28251900
O	-1.53203100	-1.95484600	-0.76442700
H	-1.52083700	-2.89894400	-0.58521900
C	-1.78465300	0.06841700	0.37438200
H	-2.84857000	0.12022500	0.54478500

1... •OH(1)			
C	0.79292200	1.74530400	-0.00847500
C	1.98598100	1.13341000	-0.00445300
H	0.67926200	2.81917400	-0.01222100
H	2.90930300	1.68949000	-0.00462400
C	2.04806700	-0.31175800	0.00108500
O	3.05133400	-0.99584800	0.00550600
C	-0.39734800	-0.29952400	-0.00416900
S	-1.86563800	-1.14992300	-0.00238700
N	0.78806100	-0.93371200	0.00059600
H	0.78767700	-1.94416900	0.00317200
N	-0.37544700	1.03205200	-0.00751500
H	-1.32995200	1.43752900	-0.00714900
O	-2.95945700	0.90958100	0.11220400
H	-3.39713000	0.79391800	-0.73817500

1... •OH(2)			
C	-1.72564700	-1.52051300	0.00159700
C	-2.32906700	-0.32568600	-0.00053800
H	-2.26048200	-2.45833900	0.00386300
H	-3.40327500	-0.23655400	0.00050600
C	-1.52722500	0.88198800	-0.00375000
O	-1.94762900	2.02202000	-0.00620800
C	0.44258200	-0.54962000	-0.00252000
S	2.13251900	-0.69607100	-0.00165500
N	-0.14855900	0.64684500	-0.00339800
H	0.53824700	1.42181700	-0.00397000
N	-0.35569200	-1.62852100	0.00075000

H	0.07686900	-2.53824600	0.00092700
O	2.26995900	1.62820900	0.10840900
H	2.71560500	1.70135400	-0.74264900

**6•**			
C	-0.77346900	1.73326400	0.00000000
C	-1.79471300	0.84176300	0.00000000
H	-0.97191300	2.79733500	0.00000200
H	-2.82709300	1.15785400	0.00000200
C	-1.51507800	-0.57811800	-0.00000200
O	-2.33888700	-1.47862900	0.00000100
C	0.81921300	0.10310500	-0.00000100
S	2.45463800	-0.41581800	0.00000000
N	-0.15766100	-0.85044500	0.00000000
N	0.54384900	1.38604000	0.00000000
H	0.11685300	-1.82232300	0.00000000

**7•**			
C	0.81895500	1.70816400	0.00000000
C	1.81272500	0.80275300	-0.00000900
H	0.97609100	2.77638300	0.00000800
H	2.84594300	1.11395200	-0.00000900
C	1.50042800	-0.61316000	-0.00001600
O	2.35448300	-1.49113400	0.00002300
C	-0.75983000	-0.03651200	-0.00000500
S	-2.45782400	-0.46072200	0.00000300
N	0.14313500	-0.96403500	-0.00001800
N	-0.47645100	1.29080400	0.00000800
H	-1.23318300	1.95542800	0.00002900

**2•+**			
C	-2.66412700	-0.50294100	1.79143000
C	-3.81961100	0.12863600	1.56208000
H	-2.38795300	-0.91401000	2.75053500
H	-4.55359000	0.26563000	2.33933100
C	-4.10428200	0.64332500	0.23969700
O	-5.09916100	1.23086600	-0.11696200
C	-1.91860700	-0.22760900	-0.44717500
S	-0.77901900	-0.42591700	-1.70660900
N	-3.07232200	0.40266900	-0.69382800
H	-3.23614800	0.74818900	-1.63011200
N	-1.72961100	-0.67416000	0.79344200
H	-0.86417500	-1.16133300	0.98905700
C	4.33961600	-0.12116700	0.87606200
C	4.16335500	1.17797600	0.61495300
H	5.22600300	-0.52309100	1.34276400
H	4.91402100	1.91115900	0.86091600
C	2.93847500	1.62185600	-0.01479400
O	2.63960500	2.75543600	-0.31185200
C	2.21906400	-0.71803600	-0.02119700
S	1.07477400	-1.93685700	-0.38306000
N	2.03053900	0.57509500	-0.28914400
H	1.16080300	0.83927900	-0.74001000
N	3.37516100	-1.05203400	0.55775800
H	3.53574100	-2.02509100	0.76880100

2•			
C	-2.56874700	-0.50671300	1.78182700
C	-3.71565000	0.15375300	1.58620200
H	-2.27690500	-0.92318800	2.73418700
H	-4.42309300	0.31009600	2.38409000
C	-4.02361200	0.67334900	0.27139700
O	-5.01692100	1.28612300	-0.05481600
C	-1.87884400	-0.25243600	-0.48125900
S	-0.77599100	-0.48983500	-1.75128500
N	-3.02854700	0.40928400	-0.68851300
H	-3.20633900	0.75940700	-1.61987800
N	-1.67041000	-0.70258600	0.76001400
H	-0.80829800	-1.21361100	0.92066500
C	4.26753700	-0.09596000	0.95206900
C	4.09425200	1.20709300	0.63049400
H	5.16237500	-0.41354300	1.47414300
H	4.82699100	1.96017000	0.87739100
C	2.90116700	1.60524300	-0.06256500
O	2.59443800	2.73471400	-0.42551600
C	2.29613700	-0.74245400	0.04305500
S	1.12702500	-1.99092200	-0.33588900
N	2.04262900	0.53845900	-0.31720600
H	1.18219000	0.74737500	-0.80895400
N	3.38103700	-1.09551900	0.66691600

2•-			
C	-2.20227800	1.72397900	0.53639800
C	-3.54064000	1.65275200	0.52861300
H	-1.66056000	2.59420500	0.87770600
H	-4.15542400	2.46987300	0.86951800
C	-4.18486700	0.44939800	0.05294100
O	-5.37885400	0.24008900	-0.01656700
C	-1.94036800	-0.45631600	-0.34158800
S	-0.97836000	-1.74717200	-0.88952600
N	-3.28282000	-0.54957300	-0.34901300
H	-3.69083700	-1.41715600	-0.66839100
N	-1.41771800	0.68727200	0.10060800
H	-0.37823200	0.78433900	0.07891900
C	2.20711500	1.74750500	-0.47966400
C	3.55319700	1.65351600	-0.54032300
H	1.70423500	2.65382400	-0.79390200
H	4.16759600	2.46528100	-0.89806300
C	4.19261300	0.43427800	-0.12481900
O	5.39122100	0.19101200	-0.11891800
C	1.93841400	-0.36476700	0.34631000
S	1.00068800	-1.69800400	0.95511100
N	3.28323800	-0.53669800	0.29224100
H	3.66994500	-1.42813500	0.56746900

1a⁻… •OH(1)			
C	-0.81553000	1.74569500	-0.01739700
C	-2.01610100	1.11595100	-0.00288500
H	-0.77611300	2.82938400	-0.02647400
H	-2.94914100	1.65838900	0.00016300
C	-2.04472400	-0.31732700	0.00988700
O	-3.03784700	-1.04140500	0.02556000
C	0.39755300	-0.18476600	-0.01295600
S	1.85218300	-1.11354700	-0.02091600
N	-0.77654300	-0.88246400	0.00350800
H	-0.73242500	-1.89076100	0.01306100
N	0.39674500	1.12878500	-0.02262100
O	3.27610400	0.79624100	0.04875100
H	2.44808600	1.29949000	0.02731600

1a⁻… •OH(2)			
C	1.67982900	-1.54899000	0.00357100
C	2.30834400	-0.34586200	0.00428000
H	2.26497000	-2.46227100	0.00534400
H	3.38490500	-0.26404700	0.00642900
C	1.51849800	0.85225200	0.00148100
O	1.93799000	2.01094300	0.00194500
C	-0.38952900	-0.63402500	-0.00067500
S	-2.12734100	-0.72321300	-0.00264200
N	0.15851800	0.60196000	-0.00214700
H	-0.54365600	1.36227600	-0.00250200
N	0.33138100	-1.73007800	0.00077200
O	-2.25383600	1.63803900	-0.09861700
H	-2.67414300	1.70016800	0.76405800

1b⁻… •OH(1)			
C	0.78616100	-1.72708500	-0.00074300
C	1.97491500	-1.09959000	0.00297200
H	0.67413200	-2.80226900	-0.00151300
H	2.90240300	-1.65173100	0.00527300
C	2.00975200	0.35329000	0.00350400
O	3.07412700	0.98331800	0.00713300
C	-0.31439500	0.34577400	-0.00260300
S	-1.84892400	1.17448900	-0.00623900
N	0.80219800	1.02619200	-0.00012800
N	-0.36351300	-1.00369400	-0.00417800
H	-1.32832600	-1.38285800	-0.00298800
O	-2.96335400	-0.92579500	-0.09043900
H	-3.36101100	-0.80697900	0.77686300

1b⁻… •OH(2)			
C	1.74016200	1.50047000	-0.00000200
C	2.33511400	0.29892100	-0.00001200
H	2.27404000	2.43985800	-0.00000400
H	3.40987900	0.20325700	-0.00002400
C	1.50836400	-0.89702600	-0.00000100
O	1.99712100	-2.03044600	0.00001100
C	-0.39695200	0.46463300	0.00000200
S	-2.10824300	0.71840500	0.00000000
N	0.13600800	-0.73129400	-0.00000400
N	0.37963800	1.58687000	0.00001000
O	-2.61643800	-1.60644200	-0.00000300
H	-1.65313900	-1.71814100	-0.00000600
H	-0.07400700	2.48463900	0.00001600

3a•- Hext			
C	0.62541600	-1.55865500	-0.03139100
C	1.57240800	-0.48431200	0.38852600
H	1.02326700	-2.55604400	-0.17518000
C	0.99728900	0.88090000	0.06988600
O	1.72828900	1.84922100	-0.09550200
C	-1.21304100	-0.17736700	-0.01508600
S	-2.89460400	0.15755300	-0.01031300
N	-0.34873900	0.93980200	0.02039500
N	-0.68743600	-1.38614800	-0.10904800
H	-0.77221500	1.84743600	-0.09479200
H	1.71159600	-0.50562200	1.48329600
O	2.82972500	-0.64540300	-0.23754700
H	3.24770300	0.22387500	-0.23496000

4a•- Hint			
C	-1.13872800	-1.17671200	0.41042200
C	-0.98287000	1.34181100	0.00228800
O	-1.45732000	2.46737400	-0.15040000
C	0.97244500	-0.14567500	0.08496300
S	2.69919800	-0.11144100	-0.07787100
N	0.36585300	1.11808600	-0.02233500
N	0.29443400	-1.21847000	0.28807100
H	0.97103700	1.90609700	-0.18305000
H	-1.41454600	-1.54580600	1.40595700
O	-1.73812700	-2.12589800	-0.47343000
H	-1.43059000	-1.92413000	-1.36220000
C	-1.77152100	0.15794800	0.21459900
H	-2.84746900	0.25354500	0.24207700

3b•-			
C	0.64745400	-1.51797100	-0.19489300
C	1.52153800	-0.49244200	0.41536400
H	0.95061000	-2.53956800	-0.35629400
C	0.97747300	0.88541300	0.02177700
O	1.80776100	1.76423700	-0.22233500
C	-1.18062500	0.03566500	-0.02985900
S	-2.88041000	0.20023400	0.07140000
N	-0.35264500	1.08406800	-0.00533100
N	-0.68682200	-1.22631800	-0.19520000
H	-1.36319000	-1.95503800	-0.35123600
H	1.46205600	-0.54990800	1.51717300
O	2.86392900	-0.62739700	0.01767600
H	3.14479400	0.27782100	-0.18537800

4b•-			
C	1.05678400	-1.25476000	-0.40203800
C	1.05954600	1.26918800	-0.00274200
O	1.71431800	2.31403600	0.14069000
C	-0.96780000	0.12864500	-0.04646200
S	-2.68306400	0.08269200	0.07928500
N	-0.29381900	1.24980100	0.18066800
N	-0.35978000	-1.02943400	-0.41360600
H	-0.96346400	-1.81747000	-0.57057900
H	1.33282400	-1.81333400	-1.30072900
O	1.47748800	-2.00425300	0.73871200
H	1.34130900	-2.93856300	0.56129400
C	1.75675600	0.04888000	-0.35947800
H	2.82738000	0.07374200	-0.49886200

8•-

C	-0.81106900	2.02834200	0.00024300
C	-1.85616000	1.08611000	-0.00005900
H	-0.92913800	3.09714800	-0.00046900
H	-2.88494000	1.41570300	-0.00022400
C	-1.59777500	-0.26129600	-0.00003300
O	-2.34757300	-1.28458900	-0.00015500
C	0.85488900	0.24762900	0.00003400
S	2.49035800	-0.23129400	-0.00009100
N	-0.17107500	-0.60437500	0.00023600
N	0.51250800	1.54539300	-0.00002100
H	1.28079300	2.19199400	-0.00001600
O	0.05367600	-1.95786200	0.00010300
H	-0.89060300	-2.25636800	-0.00001900
**10•-**			
C	1.78330800	-0.59623800	0.00000000
C	0.97681700	-1.69043300	0.00000000
H	2.86311200	-0.70442500	0.00000000
H	1.38512000	-2.69170000	0.00000000
C	-0.45413200	-1.51132900	0.00000000
O	-1.26079200	-2.46168500	0.00000000
C	0.00000000	0.74753300	0.00000000
S	-0.68148800	2.38184900	0.00000000
N	-0.90120100	-0.20825300	0.00000000
N	1.31633700	0.67722700	0.00000000
**TS6•**			
C	-0.72507900	1.73228200	-0.00338500
C	-1.93863500	1.15238500	0.00145300
H	-0.59564200	2.80532900	-0.00591400
H	-2.84698000	1.73341600	0.00292300
C	-2.04443100	-0.28807500	0.00425700
O	-3.06707100	-0.94646000	0.00899300
N	-0.80135400	-0.94235300	0.00123700
H	-0.82484300	-1.95254900	0.00344200
N	0.42916000	1.00163800	-0.00590900
H	1.57084800	1.28864000	-0.00964900
O	2.84375800	0.97409400	-0.08973300
H	3.23307500	0.95618400	0.79042800
C	0.38615000	-0.31520700	-0.00287400
S	1.86171100	-1.19721300	-0.00620700
**TS7•**			
C	1.70206000	-1.54409000	0.00614400
C	2.29504400	-0.34380200	0.00466200
H	2.24054000	-2.47981500	0.00967100
H	3.36935800	-0.25022300	0.00664500
C	1.48502900	0.86289000	-0.00030000
O	1.92548200	1.99942700	-0.00098800
C	-0.43990700	-0.55621700	-0.00107100
N	0.10946800	0.64123800	-0.00452400
H	-0.80292100	1.40856500	-0.01197000
N	0.33175800	-1.65243100	0.00332400
H	-0.10354500	-2.56065700	0.00498000
O	-2.06478200	1.69462200	-0.09632200
H	-2.41906600	1.86101100	0.78318500
S	-2.15699400	-0.68535100	-0.00389000
**TS7$_2$•**			
C	0.23739400	2.20954900	0.00545900
C	-1.05452100	1.82635900	-0.00025800
H	0.54491000	3.24489800	0.00983600
H	-1.86827000	2.53161100	-0.00039200
C	-1.29523300	0.41946300	-0.00618600
O	-2.46226000	-0.07357400	-0.00663400
C	1.02645100	-0.05232800	-0.00030400
N	-0.25861600	-0.44143600	-0.00799900
H	-0.89465800	-1.56352800	-0.01362800
N	1.24071500	1.29470700	0.00584100

H	2.20074100	1.60120500	0.01009000
O	-1.91656900	-2.15998800	-0.09435600
H	-2.28100300	-2.26690500	0.79375400
S	2.31060500	-1.12937100	0.00194400

$TS10_a^{\bullet -}$

C	1.64514200	-1.58097300	0.01107800
C	2.26637300	-0.37420000	0.00830700
H	2.22951900	-2.49478300	0.01866700
H	3.34344800	-0.29075200	0.01350400
C	1.47197900	0.82558000	-0.00257700
O	1.91979900	1.97935100	-0.00570300
C	-0.39357700	-0.63772800	-0.00396400
N	0.11327100	0.59899100	-0.00953200
H	-0.80099200	1.37539400	-0.01564700
N	0.29511200	-1.75405800	0.00466200
O	-2.02072800	1.76929000	-0.08768500
H	-2.35188400	1.91728400	0.80197000
S	-2.15067800	-0.73792900	-0.00714700

$TS10_{a2}^{\bullet -}$

C	-0.70092300	1.71504500	-0.00901100
C	-1.91635300	1.12863900	0.00046100
H	-0.56754900	2.78918300	-0.01428900
H	-2.82412200	1.71370300	0.00309700
C	-2.00692400	-0.31830000	0.00672600
O	-3.09288700	-0.91567600	0.01603100
N	-0.82049500	-1.02802900	0.00168200
N	0.43194100	0.97110900	-0.01289100
H	1.58004100	1.24339400	-0.01248000
O	2.86453300	1.02256700	-0.07883600
H	3.22429600	0.99040600	0.81132700
C	0.30378000	-0.36056300	-0.00664500
S	1.81603500	-1.26139400	-0.00974600

$TS10_b^{\bullet -}$

C	0.17937800	2.21437200	0.00791800
C	-1.12493500	1.78422900	-0.00031300
H	0.38399100	3.28040200	0.01392500
H	-1.96665400	2.45787700	0.00020400
C	-1.29245300	0.39025600	-0.00857500
O	-2.44985500	-0.18278800	-0.00878900
C	1.04528300	0.09827100	-0.00036600
N	-0.21311800	-0.40299000	-0.01103700
H	-0.76738200	-1.55993900	-0.01583800
N	1.25660900	1.42087300	0.00855800
O	-1.72424300	-2.26148200	-0.09037400
H	-2.08173100	-2.37543700	0.79665500
S	2.35478000	-1.01854300	0.00148300
C	0.17937800	2.21437200	0.00791800

$RC^{\bullet}$

N	0.17881400	1.01142000	0.03520600
C	-1.19006700	0.95776300	-0.24361600
C	-1.64385500	-0.33575800	-0.71628800
C	-0.74681100	-1.32807300	-0.87916300
N	0.57108300	-1.15130300	-0.58232200
C	1.08169400	0.01163100	-0.10447700
O	-1.87847000	1.94500200	-0.08284900
O	-1.72614600	-1.13459700	1.61355700
H	1.21962200	-1.91278200	-0.69868100
H	0.53023200	1.89282100	0.38032000
H	-2.68120100	-0.45921800	-0.98141600
H	-1.01299100	-2.30697300	-1.24963000
H	-2.57807000	-1.56609200	1.44215100

$TS4^{\bullet}$

C	0.89742700	-1.20905700	-0.75241100
C	1.16340300	1.11241000	-0.09086100

O	1.78577800	2.13234200	0.12922800
C	-1.04967500	0.02927900	-0.09982400
S	-2.68715900	0.10883600	0.17530900
N	-0.21563800	1.07322500	0.12943300
N	-0.44889900	-1.09001000	-0.58033700
H	-1.04440500	-1.88658300	-0.73743700
H	-0.63821000	1.92096300	0.47876500
H	1.23245100	-2.13342600	-1.19494600
O	1.52285900	-1.67077600	1.20633100
H	2.46526500	-1.86946800	1.10944600
C	1.72038500	-0.12763200	-0.58650900
H	2.77287100	-0.17790800	-0.81127800

$TS3^{\bullet}$

C	-1.17108900	0.91243800	-0.28153500
C	-1.62815300	-0.43413300	-0.60002600
C	-0.70699900	-1.42058900	-0.75548100
N	0.60395900	-1.20628200	-0.47810600
C	1.10612700	-0.00590900	-0.08017100
N	0.19518400	0.99598400	-0.01058500
O	-1.86312300	1.90611400	-0.23644100
H	1.26472900	-1.96277600	-0.55536600
H	0.54460400	1.90600700	0.25276700
H	-2.65226600	-0.56871000	-0.90588800
H	-0.96392800	-2.41905400	-1.07631200
O	-1.92193100	-0.84521200	1.51628100
H	-2.54231000	-1.57428400	1.37480900
S	2.71476700	0.20580300	0.27471100

$TS3_a^{\bullet -}$

C	-1.06246900	0.68644000	-0.48519600
C	-1.42726100	-0.68377900	-0.66326000
C	-0.45670700	-1.63724600	-0.50043200
N	0.82791800	-1.40445000	-0.18210200
C	1.20059300	-0.13626500	-0.02798300
N	0.27477800	0.86193900	-0.18395500
O	-1.81774800	1.66277300	-0.54328000
H	0.58784000	1.81086900	-0.04437900
H	-2.42779200	-0.93272600	-0.97928800
H	-0.71560600	-2.68262100	-0.63509400
O	-2.75762000	-0.33902200	1.47909200
H	-2.85892000	0.49945400	0.99461300
S	2.79835100	0.32110600	0.36258000

$TS3_b^{\bullet -}$

C	1.06255800	0.83155900	-0.39761500
C	1.48016000	-0.52584100	-0.72270700
C	0.56087700	-1.51425200	-0.65447000
N	-0.70699800	-1.22506600	-0.29180400
C	-1.12019800	0.06681100	-0.04084500
N	-0.25828500	1.05906400	-0.09925000
O	1.88647700	1.75681200	-0.36192500
H	-1.40142800	-1.95073800	-0.23258500
H	2.48296000	-0.71419100	-1.07100200
H	0.77449300	-2.55047600	-0.87495600
O	2.45695100	-0.67401800	1.50326500
H	2.72262500	0.21877700	1.22617900
S	-2.77934200	0.27166400	0.34080300

$TS4_a^{\bullet -}$

C	0.87648800	-1.21937500	-0.71452700
C	1.10632500	1.12260900	-0.08662200
O	1.69812200	2.17789900	0.12343800
C	-1.02457600	-0.09511900	-0.12091900
S	-2.70228200	0.05482000	0.15729600

N	-0.25130900	1.01457800	0.14220500
N	-0.46659800	-1.20723900	-0.57354800
H	-0.72516200	1.83819500	0.47919200
H	1.28755000	-2.10794200	-1.17500400
O	1.61439000	-1.62823000	1.13708600
H	2.53798700	-1.85695500	0.97412800
C	1.69470700	-0.09018100	-0.58243500
H	2.74371900	-0.10675900	-0.83283200

$TS4_b^{\bullet -}$

C	0.86540800	-1.18259700	-0.76680900
C	1.09966700	1.13643500	-0.06366100
O	1.80586900	2.12914700	0.14215500
C	-1.01118500	0.12562700	-0.07203600
S	-2.70304200	0.10516900	0.18874600
N	-0.25300300	1.17088400	0.17739400
N	-0.46191400	-1.03715500	-0.58075600
H	1.19426900	-2.10007100	-1.22860700
O	1.57636600	-1.70492800	1.15897000
H	2.51931600	-1.86318900	1.02117900
C	1.68418400	-0.09580500	-0.57480900
H	2.73754300	-0.13909100	-0.80216300
H	-1.08436400	-1.81216900	-0.73191500

$RC10_b^{\bullet -}$

C	0.87720700	2.10188100	-0.00003000
C	-0.48466700	2.06061500	-0.00000700
H	1.37909100	3.06406400	-0.00005400
H	-1.09019300	2.95364800	-0.00001100
C	-1.11581500	0.78716100	0.00002300
O	-2.34249200	0.57097100	0.00004800
C	1.13709700	-0.16723400	0.00000100
N	-0.23217100	-0.26962100	0.00002600
H	-0.62539500	-1.20134000	0.00005000
N	1.70056200	1.03014500	-0.00002700
O	-2.92204700	-2.03626200	-0.00005400
H	-2.80333000	-1.04350600	-0.00001500

$RC7^{\bullet}$

C	0.79874600	2.13700700	-0.00006100
C	-0.54062800	2.07188200	-0.00001000
H	1.34508100	3.06849900	-0.00011000
H	-1.15509600	2.95683100	-0.00001700
C	-1.17489900	0.77861400	0.00004900
O	-2.38496500	0.57878600	0.00008900
C	1.05841600	-0.24482700	0.00000800
N	-0.29743300	-0.30079100	0.00005700
H	-0.71561500	-1.22513800	0.00011300
N	1.56988800	1.00787000	-0.00005300
H	2.57417900	1.08500800	-0.00009300
O	-2.76557000	-2.12939500	-0.00012200
H	-2.83976800	-1.14175600	0.00003500
S	2.01490700	-1.60901100	0.00002500

$6^{\bullet}OH_2$

C	-0.68348700	1.73946400	-0.00651000
C	-1.93181600	1.22555500	0.00007800
H	-0.52752100	2.81072900	-0.01119800
H	-2.80869000	1.85440900	0.00090000
C	-2.11091900	-0.20218100	0.00546500
O	-3.16483200	-0.81788200	0.01185300
N	-0.89924000	-0.90100600	0.00167000
H	-0.96732400	-1.90914700	0.00250700
N	0.46222900	0.98904000	-0.00710700
H	2.31180500	1.27568000	-0.01905100
O	3.23622800	0.96664500	-0.08833500
H	3.58998600	1.01006400	0.80361100
C	0.31236000	-0.29999900	-0.00349000

S	1.71080200	-1.35156900	-0.00625700
**7·OH2**			
C	1.91922900	-1.37148900	0.00931500
C	2.35582500	-0.10532400	0.00681600
H	2.56655400	-2.23554100	0.01516300
H	3.41058800	0.12119500	0.01086500
C	1.39577700	0.98671900	-0.00209100
O	1.72251700	2.16699300	-0.00656500
C	-0.30518200	-0.60952200	-0.00157600
N	0.04562400	0.63704400	-0.00425300
H	-1.53897700	1.63008600	-0.02247500
N	0.57420500	-1.63434900	0.00368000
H	0.24803200	-2.58710100	0.00285300
O	-2.50899200	1.73081700	-0.09245600
H	-2.81300600	1.91843900	0.79901500
S	-2.00713000	-1.02817000	-0.00525200
**10_{a^-}·OH2**			
C	1.81023600	-1.46688800	0.03638400
C	2.32292900	-0.20391800	0.02124000
H	2.47004000	-2.33419800	0.06303700
H	3.39344300	-0.02436000	0.03535900
C	1.42009800	0.92857100	-0.01438100
O	1.79485900	2.11247500	-0.03052600
C	-0.27860800	-0.66101600	-0.01135000
N	0.06768100	0.61413800	-0.02971200
H	-1.43185100	1.61557900	-0.05915700
N	0.47788000	-1.74598500	0.01966900
O	-2.39990000	1.82349500	-0.04330500
H	-2.60568200	1.84677500	0.90055500
S	-2.02828000	-1.01557000	-0.02938700
C	1.81023600	-1.46688800	0.03638400
